# Effect of Short, Animated Video Storytelling on Maternal Knowledge and Satisfaction in the Perinatal Period in South Africa: Randomized Controlled Trial

**DOI:** 10.2196/47266

**Published:** 2023-10-13

**Authors:** Maya Adam, Zwannda Kwinda, Mithilesh Dronavalli, Elizabeth Leonard, Vān Kính Nguyễn, Vusani Tshivhase, Till Bärnighausen, Yogan Pillay

**Affiliations:** 1 Department of Pediatrics Stanford University School of Medicine Stanford, CA United States; 2 Institute of Global Health Heidelberg University Heidelberg Germany; 3 Center for Digital Health, Department of Medicine Stanford University School of Medicine Stanford, CA United States; 4 Clinton Health Access Initiative South Africa Pretoria South Africa; 5 Translational Health Research Institute Western Sydney University Sydney Australia; 6 Faculty of Medicine University of New South Wales Sydney Australia; 7 Department of Global Health and Population Harvard TH Chan School of Public Health Cambridge, MA United States; 8 Africa Health Research Institute Somkhele South Africa; 9 Department of Global Health Stellenbosch University Stellenbosch South Africa; 10 Bill and Melinda Gates Foundation Pretoria South Africa

**Keywords:** maternal child health, mHealth, mobile health, randomized controlled trial, short animated storytelling, South Africa, video health messaging

## Abstract

**Background:**

Innovative mobile health (mHealth) interventions can improve maternal knowledge, thereby supporting national efforts to reduce preventable maternal and child mortality in South Africa. Studies have documented a potential role for mobile video content to support perinatal health messaging, enhance maternal satisfaction, and overcome literacy barriers. Short, animated storytelling (SAS) is an innovative, emerging approach to mHealth messaging.

**Objective:**

We aimed to measure the effect of SAS videos on maternal knowledge and user satisfaction for mothers enrolled in antenatal care programs at 2 public health facilities in the Tshwane District of South Africa.

**Methods:**

We used a randomized controlled trial with a nested evaluation of user satisfaction. Participants were randomized 1:1 into Standard-of-Care (SOC) Control, and SAS Intervention groups. The intervention videos were delivered through WhatsApp, and 1 month later, participants responded to telephone surveys assessing their knowledge. The intervention group then participated in a nested evaluation of user satisfaction.

**Results:**

We surveyed 204 participants. Of them, 49.5% (101/204) were aged between 25 and 34 years. Almost all participants self-identified as Black, with the majority (190/204, 93.2%) having completed secondary school. The mean overall knowledge score was 21.92/28. We observed a slight increase of 0.28 (95% uncertainty interval [UI] –0.58 to 1.16) in the overall knowledge score in the intervention arm. We found that those with secondary education or above scored higher than those with only primary education by 2.24 (95% UI 0.76-4.01). Participants aged 35 years or older also scored higher than the youngest age group (18-24 years) by 1.83 (95% CI 0.39-3.33). Finally, the nested user satisfaction evaluation revealed high maternal satisfaction (4.71/5) with the SAS video series.

**Conclusions:**

While the SAS videos resulted in high user satisfaction, measured knowledge gains were small within a participant population that was already receiving perinatal health messages through antenatal clinics. The higher knowledge scores observed in older participants with higher education levels suggest that boosting maternal knowledge in younger mothers with lower education levels should continue to be a public health priority in South Africa. Given the high maternal satisfaction among the SAS video-users in this study, policy makers should consider integrating similar approaches into existing, broad-reaching perinatal health programs, such as MomConnect, to boost satisfaction and potentially enhance maternal engagement. While previous studies have shown the promise of animated video health education, most of this research has been conducted in high-income countries. More research in underresourced settings is urgently needed, especially as access to mobile technology increases in the Global South. Future studies should explore the effect of SAS videos on maternal knowledge in hard-to-reach populations with limited access to antenatal care, although real-world logistical challenges persist when implementing studies in underresourced South African populations.

**Trial Registration:**

Pan African Clinical Trials Registry PACTR202203673222680; https://tinyurl.com/362cpuny

## Introduction

Despite documented reductions in maternal and child mortality during the era of the Millennium Development Goals [1-3], millions of women and babies still die each year in South Africa from largely preventable perinatal complications [4,5]. An important component of South Africa’s efforts to achieve the 2030 Sustainable Development Goals lies in improving maternal knowledge by increasing access to critical health information in the perinatal period [5,6]. Evidence-based health information and recommendations that motivate life-saving health behaviors often fail to reach mothers because they are too technical or because they fail to engage mothers in ways that optimize user satisfaction during the potentially stressful perinatal period [5,7,8]. Mobile health (mHealth) interventions have shown promise toward broadly disseminating perinatal messages and engaging expectant mothers. As part of their efforts to improve maternal-child health outcomes, the South African National Department of Health introduced an mHealth intervention named “MomConnect” in 2014 [9]. Within 3 years, the program had reached more than 1.5 million pregnant women, sending them free, twice-weekly health information SMS text messages [10]. The impressive reach of MomConnect has been documented and supports an increasingly relevant role for mHealth interventions in perinatal care protocols [11].

SMS text message–based dissemination was originally chosen to make MomConnect universally accessible to mothers with basic (nonsmart) cell phones [10]. Previous research has documented the broad reach and positive maternal experiences of using the program, suggesting that these could be further enhanced by supporting its accessibility for those who struggle to read or write [12]. In a qualitative study of mothers in rural areas of South Africa, the desire for integrated videos or voice recordings to support health messages [13] also emerged as a theme. The increasing availability of smartphones in South Africa [10,14] presents new opportunities to enhance initiatives like MomConnect by integrating innovative approaches, like animated video storytelling, to increase accessibility, ease of use, engagement, and user satisfaction.

Short, animated storytelling (SAS) videos constitute a promising new approach to public health education that emerged during the COVID-19 pandemic. The development of this approach was catalyzed by the necessity for rapid, accessible global communication of critical health messages [15,16]. The use of visual storytelling greatly increased the accessibility of health messages across languages, education levels, and literacy levels [17]. Subsequent research suggested high engagement and measurable shifts in behavioral intent in response to health messages delivered through narrated videos using a SAS approach [18,19].

Animation has been shown to support patient education [20]. Animated characters also have the potential to resonate across diverse cultural groups [21] which make up the rich cultural tapestry of South Africa. Simple, vector animations can also be reduced to small, readily shareable files that can be disseminated on popular platforms like WhatsApp [22]. “Glocalizable” characters, those that can resonate cross-culturally, also facilitate the easy translation of SAS videos into other languages simply by changing the audio tracks [21].

In this study, named the “Amandla Mama” study [23], we explore the effect of SAS videos, aligned with MomConnect messaging, on maternal knowledge and maternal satisfaction in the perinatal period. In the South African languages of isiZulu and isiXhosa, Amandla Mama translates into “motherly strength” or “power to the mother,” reflecting the overarching goals of our intervention.

The specific aims of this study are: (1) to measure the effect of watching the Amandla Mama SAS video series on knowledge of maternal and neonatal health, and (2) to measure user satisfaction associated with the Amandla Mama SAS video series.

## Methods

### Intervention

**Figure 1:**
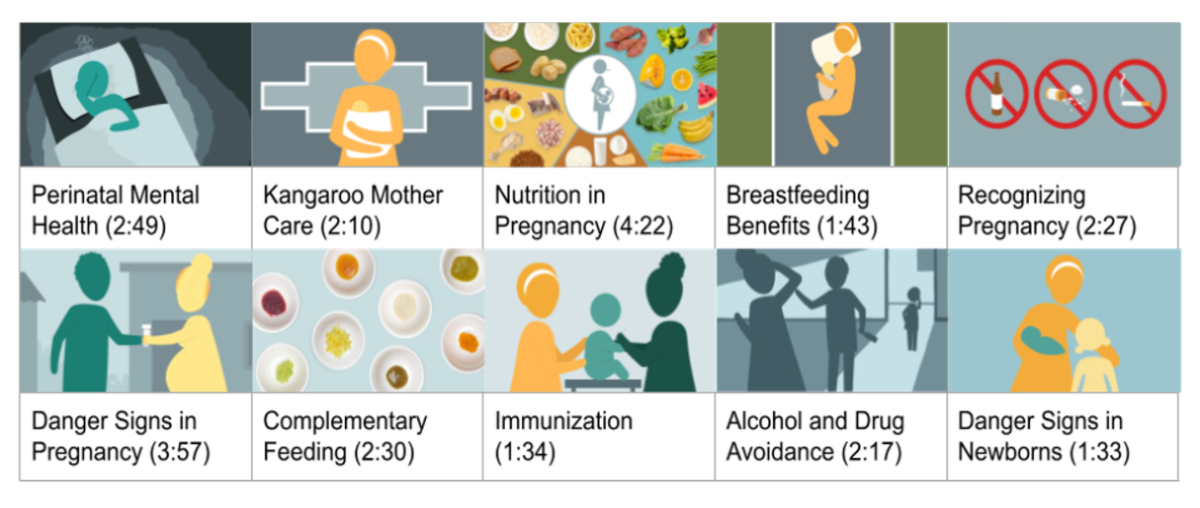
Topics included in the Amandla Mama short, animated storytelling (SAS) video intervention.

With input from local mothers and community health workers in South Africa, we developed the Amandla Mama intervention, a collection of 10 short, animated storytelling videos focused on critical perinatal health topics. During the content creation process, research partners at the Clinton Health Access Initiative in South Africa gathered formative feedback from women attending antenatal clinic visits. These expectant mothers shared their thoughts on a collection of prototypes, originally developed by our coinvestigator (MA) and the Digital Medic health education program, based in South Africa and the United States [24]. During the development of the prototypes, we received input from community health workers employed by the DG Murray Trust, a community health organization in South Africa. Using a collaborative, human-centered design process [22], mothers were asked to give feedback on the topics, style, length, narratives, audio, and visual design of the prototypes. This feedback was then used to design the final 10 intervention videos, animated by a local South African animator. Previous research on global preferences for animated character design was also used to inform the final intervention videos [21]. The topics covered in the series are described in Figure 1*,* along with screenshots and the duration of each SAS video. The videos were delivered through WhatsApp to participants in this study, and each video could be viewed only once to prevent sharing between mothers in the intervention and control groups.

### Trial Design

We used a randomized controlled trial with a nested user satisfaction evaluation to measure the effect of the Amandla Mama videos on maternal knowledge and to assess maternal satisfaction. Participants were randomly assigned to either the SAS Intervention group or the Standard-of-Care (SOC) Control group. SOC in South Africa includes regular antenatal counseling at the local clinic, according to the National Department of Health’s Guidelines for Maternity Care in South Africa [25]. This care is free of charge for expectant mothers in South Africa, and it is considered part of primary health care. As an adjunct to this maternity care, all pregnant women who register at a maternity clinic in South Africa are offered optional enrollment in MomConnect, a free service that offers informational text messages among other resources for pregnant women. In addition to having access to regular antenatal care, mothers assigned to the SAS Intervention group were sent the 10 SAS intervention videos on WhatsApp.

### Study Setting

This study took place in the City of Tshwane Metropolitan District of Gauteng, South Africa. The Tshwane Health District is situated in the northern region of Gauteng Province. In 2019, the Tshwane District had a population of more than 3 million people, with a population density of 515/km^2^ [26]. The district has 7 regions and 68 clinics that serve the 7 regions within the district. Addressing high rates of low-birth-weight babies by encouraging attendance at antenatal clinics has been a public health priority in the region [26]. Participants in this study were recruited during attendance at 2 public clinics in the City of Tshwane Metropolitan District. The Dr Fabian and Ms Florence Ribeiro Clinic is an urban community clinic located in the central business district of Tshwane, and the Kgabo Clinic is situated in the large settlement of Winterveld and serves a semirural community.

### Recruitment and Informed Consent

Participants were recruited at the clinics while they waited for their antenatal appointments. As such, this was a convenience sample, recruited consecutively by research team members affiliated with the Clinton Health Access Initiative in South Africa. Members of the research team described the trial to mothers and gained informed consent for participation in the study from mothers who expressed an interest in taking part. During recruitment, participants were notified that their participation in the study was voluntary and that they could withdraw at any time without any threat or punitive measures. They were also notified that they would receive airtime vouchers to compensate them for their time and expenses incurred by participating in the study.

### Participants and Eligibility Criteria

**Figure 2:**
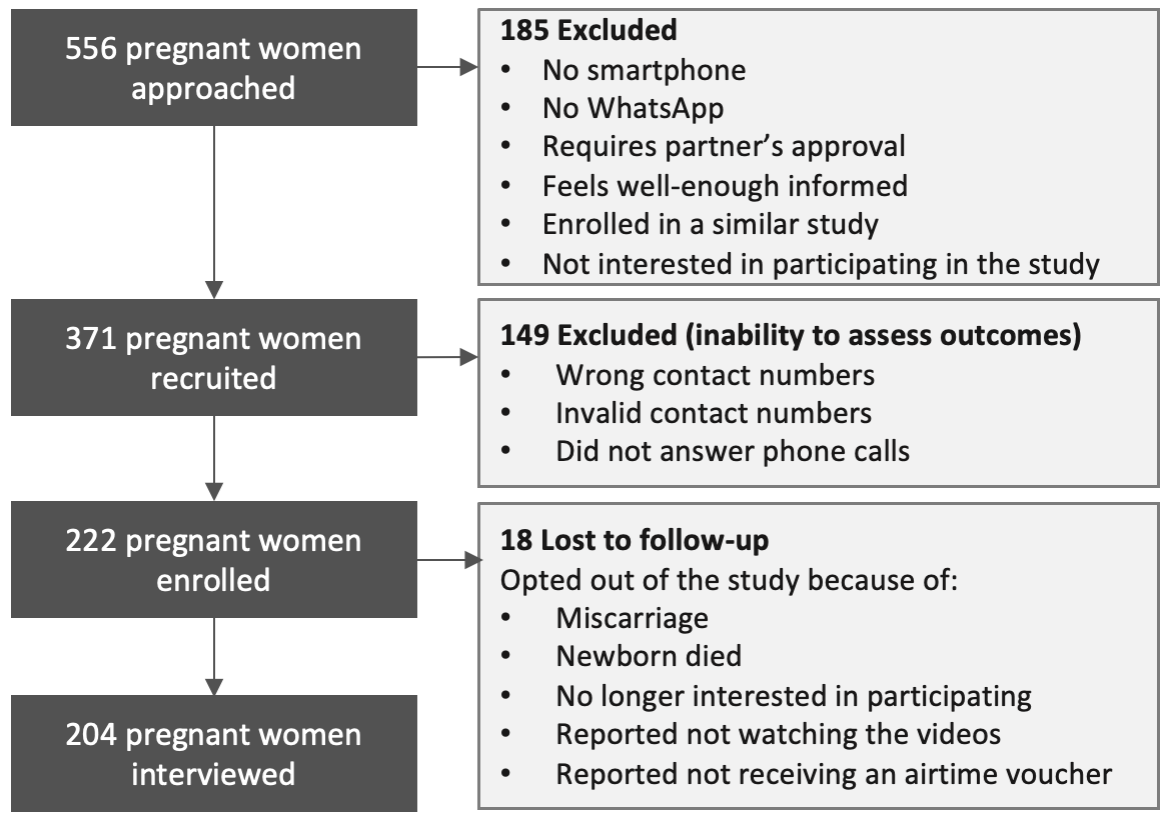
Flow diagram for the Amandla Mama Study.

Eligible participants were expectant mothers accessing routine antenatal health care services at the 2 facilities [23]. A total of 556 pregnant women were approached and screened for eligibility. Of these, 185 were excluded because of barriers that would impact their exposure to the intervention (ie, lack of reliable access to a cellphone or WhatsApp), leaving 371 who met preliminary eligibility criteria. Of these, 149 participants were excluded when attempts to reach them by phone failed because of invalid or inaccurate telephone numbers, making it impossible for them to take part in the outcomes assessment. Of the 222 enrolled participants who we were able to reach at the 2-week time point, 18 opted not to be interviewed due to the following reasons: (1) miscarriage; (2) their newborn had died; (3) they were no longer interested; or (4) they reported not having watched the videos and therefore did not want to answer questions related to them. Ultimately, we were able to interview 204 participants, and our analyses are conducted with that data. Figure 2 summarizes the flow of the trial, including the points of exclusion and loss to follow-up.

### Measures

Sociodemographic data were collected verbally from enrolled participants and immediately entered into a shared spreadsheet by our local research team. Since the trial involved testing and implementing a new intervention and we found no appropriate perinatal knowledge assessments that had been validated within this study population, the research team designed a survey consisting of 28 knowledge questions based on the intervention (Table S1 in Multimedia Appendix 1). For each knowledge question, mothers could answer “true,” “false,” or “I don’t know.” The questions were scored by allocating 1 point for each correct answer and 0 points if participants answered incorrectly or if they answered, “I don’t know.” The overall knowledge score was calculated by summing the total of the individual question scores for the 28 survey questions. Surveys were administered by follow-up telephone calls 1 month after the participants had been enrolled in the study. The participants in the intervention group were asked additional questions about their experiences with downloading and viewing the SAS video series. In order to quantify user satisfaction, we used Kano’s [27] user experience model to develop a survey measuring maternal satisfaction with the SAS video content. Participants in the intervention group were asked to respond to 8 user experience items, ranking their level of satisfaction on a scale of 1 to 5, where 1 is “not at all satisfied” and 5 is “extremely satisfied.” The adapted user satisfaction survey is shown in Textbox 1.

**Maternal satisfaction items. For each question, participants indicated how satisfied they were with the videos they saw on a scale of score 1-5, with 1=not at all satisfied and 5=extremely satisfied.**

**Questions**

1. Were the videos easy to understand? (1=not easy at all, 5=very easy)
2. Do you think the videos were useful? (1=not useful at all, 5=very useful)
3. How easy was it for you to download the videos? (1=not easy at all, 5=very easy)
4. How fast was it to download the videos? (1=very slow, 5=very fast)
5. How easy was it for you to watch the videos? (1=not easy at all, 5=very easy)
6. How easy was it for you to identify with the characters in the animated videos? (1=not easy at all, 5=very easy)
7. How likely are you to follow the advice offered in the videos? (1=not likely at all, 5=very likely)
8. How strongly do you feel that the videos gave you important information in a short time? (1=not at all, 5=very strongly)

### Outcomes

The primary outcome in this study was maternal knowledge. The secondary outcome was maternal satisfaction, defined as the mean response of all participants in the SAS Intervention group for each of the 8 satisfaction items.

### Assignment of Interventions, Allocation, and Blinding

Participants were randomly assigned 1:1 to either the SAS Intervention group or the SOC Control group. A computer-generated randomization sequence was used to assign participants to their respective groups. To implement the randomization, a table with the randomized sequence was created, and participants were added sequentially as they were enrolled in the study. The randomization sequence was masked and applied to the table by a member of the research team who was not involved in recruiting or interviewing participants. In this way, participants were allocated equally to either the control or intervention groups without the recruiters or interviewers being aware of the group to which individual participants had been assigned. In this way, both the recruiters and interviewers remained blinded to the allocation throughout the study. After the knowledge survey had been administered, the interviewers were unblinded and able to administer the maternal satisfaction questionnaire, focused on experiences using the videos, to the SAS Intervention mothers.

### Participant Time Line

Upon enrolling in the study, participants in the intervention group received the video intervention through WhatsApp, delivered to their cellphones. One month later, all of the participants were contacted telephonically by the research team to respond to the surveys. Following the unblinding of the interviewers, participants in the intervention group were asked if they had watched the videos after receiving them, and participants in the control group were asked if they had been shown any short, animated maternal health videos on the phones of others. These questions allowed us to detect any contamination in the control group and also facilitated an analysis based on the intention to treat.

### Sample Size

We determined the sample size for this trial based on our primary outcome, maternal knowledge. We used a significance level of .05 (*P*<.05) and a power of 0.9, with a 2-tailed test for significance.

We hypothesized that, at the 2-week data collection time point, the mean score of participants in the control group on the knowledge questionnaire would be 43% (12 points out of 28 points in the survey), while the mean score of the intervention group on this questionnaire would be 61% (17 out of 28 points). This would constitute an 18% absolute increase and a 1.41-times relative increase. We calculated a sample size of n=140 for each study arm, then added 50% to account for participant dropouts and loss to follow-up. This brought our sample size to n=210 for the intervention group and n=210 for the control group. Thus, we estimated a total sample size of N=420 participants for the trial. We carried out these sample size calculations on G-Power 3.1.9.6, based on the difference between 2 independent proportions, using a *z* test (power=0.9; α=.05; allocation n_1_/n_2_=1; p_1_=0.43, p_2_=0.61). Finally, we rounded up the sample size per group to the nearest 10 patients (n_1_=140, n_2_=140) [23].

### Statistical Analysis

We compared the distribution of demographic variables between the trial arms with the chi-square test or the exact Fisher test, as appropriate. Regarding the primary outcome, the sum of the scores is a discrete count, but with a bounded value at the total number of questions (28 knowledge questions). Thus, we modeled the total score using a Beta-Binomial distribution, that is, score∼BB (n=28,μ,σ), which accounts for potential overdispersion and the bounded property of the distribution. Compared to the empirical distribution of the total score, the distribution fitted better than the common count distributions, including Poisson and Negative Binomial distribution (Figure S1 in Multimedia Appendix 1).

There were 11 individuals with missing data in one of the 28 knowledge questions; thus, we used multiple imputations to impute the missing values, conducted analyses on 100 imputed samples and reported the pooled analyses. We conducted multivariable regression analyses to explore the effect on the knowledge scores of the SAS Intervention group based on intention to treat. The model was adjusted for age, sex, education level, living area, working status, and whether the mother was enrolled in the MomConnect program. The models were fitted in a Bayesian inference framework using the statistical program R (version 4.0.2; The R Foundation). We reported the 95% uncertainty interval (UI) of the mean differences between the covariates by drawing 1000 samples from the posterior distribution and calculating the quantiles of the mean difference, which is nμ for the BB distribution. (The code for analyses is publicly available at GitHub [28]). We analyzed the maternal satisfaction data by calculating a mean score for each of the different subjective user satisfaction questions to capture maternal satisfaction related to the use of the SAS videos. Finally, we measured the internal reliability of the maternal satisfaction items using a Cronbach α test.

### Access to the Full Protocol, Participant-Level Data, and Statistical Code

The protocol for this trial was published as open access in June 2022 [23], and requests to access participant-level data as well as statistical code may be sent to the Clinton Health Access Initiative in South Africa [29].

### Ethical Considerations

Ethical approval for the study was granted by the Pharma-Ethics Independent Research Ethics Committee, the Tshwane Health Research and Ethics Committee, and the Scientific Research Ethics Committee of the Clinton Health Access Initiative. We also registered this study on the National Health Research Database (GP202111071). The study researchers were trained in good clinical practice through accredited health research ethics training programs. Written informed consent was obtained from all participants.

## Results

### Participant Demographics

The participants included in our final data set were 204 pregnant women. Most (101/204, 49.5%) were aged between 25 and 34 years, and 93.2% (190/204) of them had completed secondary school. Almost all participants (201/204, 98.5%) self-identified as Black or African and the majority (169/204, 83%) were South African citizens, with the remaining 17% (35/204) of them having citizenship in other African nations, commonly Zimbabwe. We observed no significant differences in participant demographic characteristics between the 2 study arms. Table 1 summarizes the demographics of our trial participants.

**Table 1:** Demographic and baselines distribution between the 2 intervention arms.

Demographic			Intervention (video)				*P* value^a^	
			No, n (%)		Yes, n (%)			
**Age (years)**								.37^b^
	18-24	34 (0.44)		43 (0.56)				
	25-34	48 (0.47)		54 (0.53)				
	>35	14 (0.61)		9 (0.39)				
**Education**								.46^b^
	Primary school	5 (0.38)		8 (0.62)				
	Secondary and above	93 (0.49)		97 (0.51)				
**Area**								.95^b^
	Rural	30 (0.48)		32 (0.52)				
	Urban	68 (0.48)		74 (0.52)				
**Race**								.48^c^
	Black or African	96 (0.48)		106 (0.52)				
	Colored	1 (1.00)		0 (0.00)				
**Nationality**								.46^b^
	South African	81 (0.48)		89 (0.52)				
	Others	17 (0.55)		14 (0.45)				
**Work**								.25^b^
	No	58 (0.45)		71 (0.55)				
	Yes	40 (0.53)		35 (0.47)				
**Earning**								.32^b^
	Low income (US $1-$265)	8 (0.40)		12 (0.60)				
	Middle income (US $266-$1060)	7 (0.64)		4 (0.36)				
	Not willing to disclose	25 (0.58)		18 (0.42)				
**Partner**								.26^c^
	No	5 (0.71)		2 (0.29)				
	Yes	93 (0.47)		104 (0.53)				
**MomConnect**								.97^b^
	No	22 (0.48)		24 (0.52)				
	Yes	76 (0.48)		82 (0.52)				

^a^*P* value <.05 is considered statistically significant.

^b^2-tailed chi-square test.

^c^2-tailed Fisher test.

### Knowledge Outcomes

**Figure 3:**
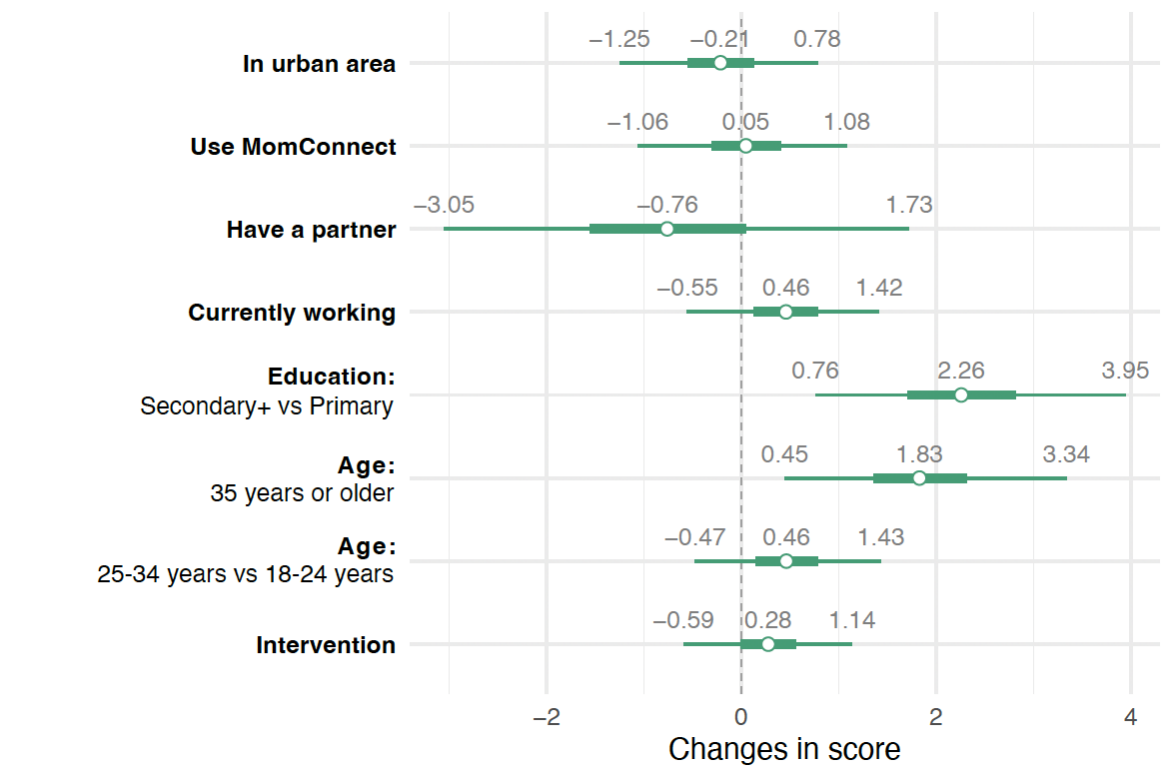
Changes in overall knowledge scores by baseline variable and intervention exposure.

Our participants achieved a mean overall score of 21.92 (78.3%) on the knowledge questionnaire (Table S1 in Multimedia Appendix 1). The results of the regression model of overall knowledge scores showed that, after adjusting for demographic variables, watching the short videos slightly increased the overall knowledge score by an average of 0.28 (95% UI –0.58 to 1.16). We found that those with education of secondary level or higher scored higher than those with primary education level by 2.24 (95% UI 0.76-4.01). Participants aged 35 years or older also scored higher than the youngest age group (18-24 years) by 1.83 (95% UI 0.39-3.33). Figure 3 shows the changes in the overall knowledge scores by baseline variable and exposure to the intervention. The points indicate the posterior median, while the thick and thin lines denote the IQR and 95% UI, respectively.

### Maternal Satisfaction Outcomes

**Figure 4:**
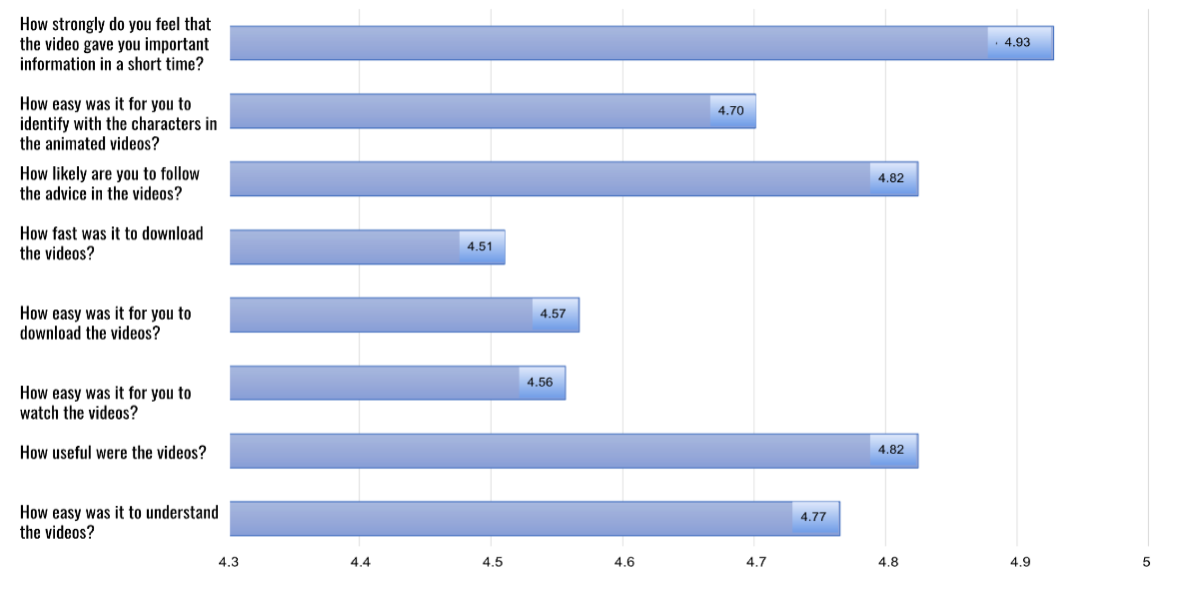
Maternal satisfaction items describing the experience of using short, animated storytelling (SAS) videos.

Finally, we present the results of our nested maternal satisfaction evaluation in Figure 4. The value for Cronbach α for the 8 questions was α=.78 (95% CI 0.71-0.84) indicating acceptable to good internal consistency. Maternal satisfaction was high overall, with a pooled average satisfaction score of 4.71 on a 5-point Likert scale. As shown in Figure 4, the lowest-scoring questions still exceeded 4.5 out of 5 and had to do with technical difficulties, including the speed and ease of downloading as well as the ease of viewing videos on mobile devices. Probing from the interviewers revealed that internet access can still be challenging in many communities, especially given frequent load-shedding, in which entire neighborhoods may have no electricity (and therefore no internet) for several hours each day. In addition, mobile devices are often shared within a family (or are the property of the husband), and finding a quiet, private location in which to view the videos could present logistical issues for some participants.

## Discussion

### Principal Results

In this study, we observed slight gains in maternal knowledge with large uncertainty after a single exposure to a series of 10 short, animated storytelling videos delivered through WhatsApp to expectant mothers in the Tshwane District of Gauteng Province, South Africa. Mothers with secondary level education or higher demonstrated higher scores than those with primary education level, and participants aged 35 years or older scored higher than the youngest age group (18-24 years). Results of our nested maternal satisfaction evaluation revealed very high overall satisfaction with the experience of using the SAS video series.

### Limitations

Our initial expectation of a larger effect size (5 points increase) led to an underpowered sample size to detect smaller gains in knowledge. Our participants were all receiving routine antenatal care, where they were likely hearing similar perinatal health messages to the ones included in the SAS videos. Other significant limitations of this study were the real-world challenges associated with testing a mHealth intervention in a setting where internet access can be unreliable, the turnover of cell phones and contact numbers is high, and mothers may be sharing cell phones with their partners. This led to a larger-than-anticipated loss to follow-up in this study and negatively impacted our ability to detect an effect of the SAS intervention. We also did not collect data on the parity of this study’s participants. Finally, we were constrained by the method of measuring knowledge in this study. Due to literacy barriers, the knowledge survey had to be administered verbally by our research assistants. The inability to administer the knowledge surveys without human intervention (ie, digitally) meant that we could not exclude the possibility that interviewers collecting survey data may have inadvertently helped the participants. This potential limitation is supported by the relatively high knowledge scores we observed across all participants.

### Comparisons With Previous Work

In a recent meta-analytic review, Feeley et al [20] documented the potential for animated videos to boost patient knowledge, but all of the studies included in the review were conducted in high-income countries, mostly in the United States. The capacity to precisely measure changes in knowledge can be constrained by the real-world limitations of conducting community-based research in underresourced settings like ours. The lack of knowledge assessment tools validated within this participant population also poses a challenge and could certainly explain our failure to document a significant change in knowledge.

### Conclusions

The high maternal satisfaction related to the use of SAS videos suggests that there may be a role for SAS videos in augmenting widely adopted interventions like MomConnect. Especially as internet access and smartphone penetration increase across South Africa, there may be a growing role for innovative approaches that encourage engagement with perinatal health services through high-satisfaction, easily scalable add-ons, like SAS videos.

While policy makers in South Africa continue to promote readily scalable interventions like MomConnect, they should consider enhancing such programs through the addition of content-aligned interventions like the Amandla Mama SAS video series to enhance user satisfaction and potentially boost engagement with perinatal health messages. Furthermore, for mothers who lack access to maternity clinics, the provision of MomConnect and SAS videos may be an effective way to transmit critical perinatal health messages to hard-to-reach communities in ways that overcome education and literacy barriers. Because of the “glocalizable” character designs used in the Amandla Mama series, these videos could also easily and cost-effectively be adapted for other language groups in South Africa and other countries. Health promotion agencies in South Africa might even consider translating and dubbing short, animated video content to make it accessible for all South African mothers—a potentially powerful step toward overcoming both language and literacy barriers to maternal health education.

## Appendix

Multimedia Appendix 1 Empirical density distribution of the total knowledge score and Maternal Knowledge Survey.

Multimedia Appendix 2 Perinatal mental health.

Multimedia Appendix 3 Nutrition in pregnancy.

Multimedia Appendix 4 Kangaroo Mother Care.

Multimedia Appendix 5 Recognizing pregnancy.

Multimedia Appendix 6 Alcohol avoidance.

Multimedia Appendix 7 Benefits of breastfeeding.

Multimedia Appendix 8 Danger signs in the newborn period.

Multimedia Appendix 9 Complementary feeding.

Multimedia Appendix 10 Immunizations.

Multimedia Appendix 11 Danger signs in pregnancy.

Multimedia Appendix 12 CONSORT-eHEALTH checklist (V 1.6.1).

## Abbreviations

mHealth: mobile health
SAS: short, animated storytelling
SOC: standard-of-care
UI: uncertainty interval

## Data Availability

The data collected as part of this study are available upon request from the corresponding author MA. The data are not publicly available, as we wish to protect the privacy of the expectant mothers who agreed to participate. The entire video series is available in Multimedia Appendices 2-11 and Google Drive [30]. These videos can be freely used by community health organizations for health promotion purposes.
