# Supplementary material for: Effect of Short, Animated Video Storytelling on Maternal Knowledge and Satisfaction in the Perinatal Period in South Africa: Randomized Controlled Trial
Source: J Med Internet Res. 2023 Oct 13;25:e47266. doi: 10.2196/47266 (PMC10612008; doi:10.2196/47266)
Supplement: Multimedia Appendix 1 [file jmir_v25i1e47266_app1.docx]

**Figure S1**. Empirical density distribution of the total knowledge score.


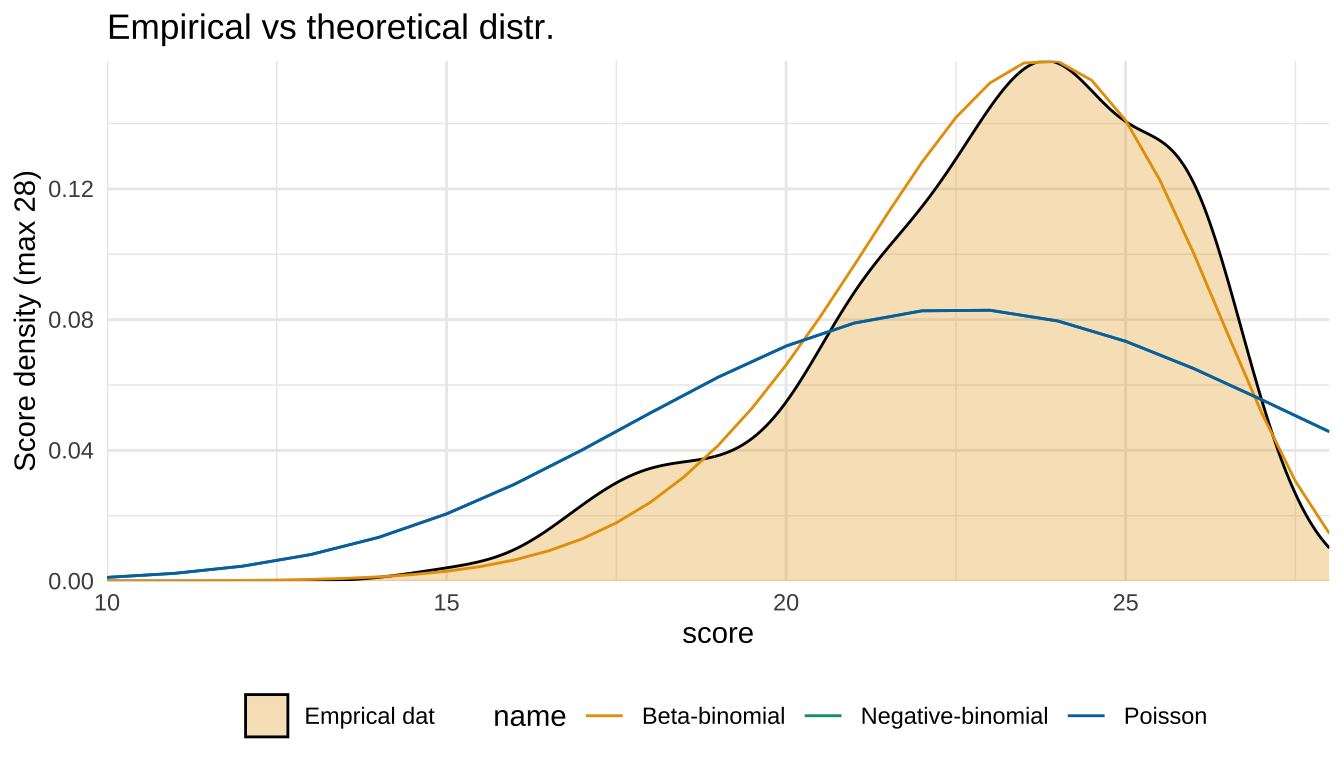


** Empirical density distribution of the total knowledge score (shaded area with black line) versus fitted distribution density of the Poisson (blue line), Negative Binomial (light blue line – overlapping with Poisson distribution), and Beta-Binomial distribution (orange line).*

**Table S1**. Amandla Mama Maternal Knowledge Survey

| **Amandla Mama Maternal Knowledge Survey**  *Instructions: For each item below, please indicate whether each statement is true or false.  You can also answer “I don’t know” if you are unsure.* | |
| --- | --- |
| *1* | *Kangaroo mother care should be avoided because it makes breastfeeding difficult* |
| *2* | *Drinking breastmilk prevents infections in a baby* |
| *3* | *Breastmilk alone provides enough nutrition until babies are 6 months old* |
| *4* | *Babies should be given water or cooled tea in addition to breastmilk when it is hot outside* |
| *5* | *Formula feeding is healthier for babies than breastfeeding if mothers can afford it* |
| *6* | *Babies under six months should be given muti if recommended by a Sangoma* |
| *7* | *Pregnant mothers should avoid fruits because it will be too acidic for the baby* |
| *8* | *It’s okay to drink alcohol when pregnant because it relaxes the mother* |
| *9* | *Iron is found in green vegetables as well as meat* |
| *10* | *It is important for pregnant women to get enough nutrients in their meals* |
| *11* | *Beans are an excellent source of protein* |
| *12* | *Eggs are an excellent source of starch* |
| *13* | *Women don’t need to take special care of their health during pregnancy* |
| *14* | *Pregnant women should visit the clinic regularly to check for infections that can affect the baby* |
| *15* | *A pregnant woman should not eat healthy food if the baby is growing well* |
| *16* | *Vaccines help to protect newborn babies from getting sick* |
| *17* | *Vaccines are dangerous for newborn babies and should be delayed until the child is older* |
| *18* | *Difficulty sleeping is during late pregnancy is a danger sign* |
| *19* | *Severe headaches during late pregnancy are danger signs* |
| *20* | *Feeling your baby kick is normal during late pregnancy* |
| *21* | *Bleeding from the vagina is normal during late pregnancy* |
| *22* | *When a newborn baby loses some weight in the first week it is a danger sign* |
| *23* | *When a newborn has a fever in the first week it is a danger sign* |
| *24* | *When a newborn cries a lot at night, it is a danger sign.* |
| *25* | *When a newborn has black stools in the first week, it is a danger sign* |
| *26* | *Pregnant women should visit the clinic regularly, even if they feel fine* |
| *27* | *Pregnant women should exercise regularly* |
| *28* | *Newborn babies can be given porridge to help them gain weight and sleep better.* |
