# Supplementary material for: Effect of Short, Animated Video Storytelling on Maternal Knowledge and Satisfaction in the Perinatal Period in South Africa: Randomized Controlled Trial
Source: J Med Internet Res. 2023 Oct 13;25:e47266. doi: 10.2196/47266 (PMC10612008; doi:10.2196/47266)
Supplement: Multimedia Appendix 12 [file jmir_v25i1e47266_app12.pdf]

CONSORT-EHEALTH (V 1.6.1) - Submission/Publication Form

The CONSORT-EHEALTH checklist is intended for authors of randomized trials evaluating web-based and Internet-based applications/interventions, including mobile interventions, electronic games (incl multiplayer games), social media, certain telehealth applications, and other interactive and/or networked electronic applications. Some of the items (e.g. all subitems under item 5 - description of the intervention) may also be applicable for other study designs.

The goal of the CONSORT EHEALTH checklist and guideline is to be
a) a guide for reporting for authors of RCTs,
b) to form a basis for appraisal of an ehealth trial (in terms of validity)

CONSORT-EHEALTH items/subitems are MANDATORY reporting items for studies published in the Journal of Medical Internet Research and other journals / scientific societies endorsing the checklist.

Items numbered 1., 2., 3., 4a., 4b etc are original CONSORT or CONSORT-NPT (non-pharmacologic treatment) items.
Items with Roman numerals (i., ii, iii, iv etc.) are CONSORT-EHEALTH extensions/clarifications.

As the CONSORT-EHEALTH checklist is still considered in a formative stage, we would ask that you also RATE ON A SCALE OF 1-5 how important/useful you feel each item is FOR THE PURPOSE OF THE CHECKLIST and reporting guideline (optional).

Mandatory reporting items are marked with a red *.
In the textboxes, either copy & paste the relevant sections from your manuscript into this form - please include any quotes from your manuscript in QUOTATION MARKS,
or answer directly by providing additional information not in the manuscript, or elaborating on why the item was not relevant for this study.

YOUR ANSWERS WILL BE PUBLISHED AS A SUPPLEMENTARY FILE TO YOUR PUBLICATION IN JMIR AND ARE CONSIDERED PART OF YOUR PUBLICATION (IF ACCEPTED).
Please fill in these questions diligently. Information will not be copyedited, so please use proper spelling and grammar, use correct capitalization, and avoid abbreviations.

DO NOT FORGET TO SAVE AS PDF _AND_ CLICK THE SUBMIT BUTTON SO YOUR ANSWERS ARE IN OUR DATABASE !!!

Citation Suggestion (if you append the pdf as Appendix we suggest to cite this paper in the caption):
Eysenbach G, CONSORT-EHEALTH Group
CONSORT-EHEALTH: Improving and Standardizing Evaluation Reports of Web-based and Mobile Health Interventions
J Med Internet Res 2011;13(4):e126

URL: http://www.jmir.org/2011/4/e126/
doi: 10.2196/jmir.1923
PMID: 22209829

madam@stanford.edu Switch account

Not shared

Draft not saved

* Indicates required question

Your name *

First Last

Maya Adam

Primary Affiliation (short), City, Country *

University of Toronto, Toronto, Canada

Stanford University, Stanford CA, USA

Your e-mail address *

abc@gmail.com

madam@stanford.edu

Title of your manuscript *

Provide the (draft) title of your manuscript.

Effect of Short, Animated Video Storytelling on Maternal Knowledge and Satisfaction in the Perinatal Period in South Africa: Randomized Controlled Trial

Name of your App/Software/Intervention *

If there is a short and a long/alternate name, write the short name first and add the long name in brackets.

Amandla Mama video series (not integrated in

Evaluated Version (if any)

e.g. "V1", "Release 2017-03-01", "Version 2.0.27913"

We did not "release" these videos. We hope the

Language(s) *

What language is the intervention/app in? If multiple languages are available, separate by comma (e.g. "English, French")

English

URL of your Intervention Website or App

e.g. a direct link to the mobile app on app in appstore (itunes, Google Play), or URL of the website. If the intervention is a DVD or hardware, you can also link to an Amazon page.

https://drive.google.com/drive/folders/1tr7h4sSDR8A5oqzvNtuWbbHX-5LG_Zkm?usp=drive_

URL of an image/screenshot (optional)

Your answer

Accessibility *

Can an enduser access the intervention presently?

- ○ access is free and open
- ○ access only for special usergroups, not open
- ○ access is open to everyone, but requires payment/subscription/in-app purchases
- ○ app/intervention no longer accessible
- ◉ Other: We did not "release" these videos. We hope they will be adopted and ir

Primary Medical Indication/Disease/Condition *

e.g. "Stress", "Diabetes", or define the target group in brackets after the condition, e.g. "Autism (Parents of children with)", "Alzheimers (Informal Caregivers of)"

Perinatal health

Primary Outcomes measured in trial *

comma-separated list of primary outcomes reported in the trial

Knowledge, Maternal Satisfaction

Secondary/other outcomes

Are there any other outcomes the intervention is expected to affect?

No

Recommended "Dose" *

What do the instructions for users say on how often the app should be used?

- ◯ Approximately Daily
- ◯ Approximately Weekly
- ◯ Approximately Monthly
- ◯ Approximately Yearly
- ◯ "as needed"
- ◉ Other: We did not "release" these videos. We hope they will be adopted and ir

Approx. Percentage of Users (starters) still using the app as recommended after 3 months *

- ◯ unknown / not evaluated
- ◯ 0-10%
- ◯ 11-20%
- ◯ 21-30%
- ◯ 31-40%
- ◯ 41-50%
- ◯ 51-60%
- ◯ 61-70%
- ◯ 71%-80%
- ◯ 81-90%
- ◯ 91-100%
- ◉ Other: We did not "release" these videos. We hope they will be adopted and ir

Overall, was the app/intervention effective? *

- ○ yes: all primary outcomes were significantly better in intervention group vs control
- ◉ partly: SOME primary outcomes were significantly better in intervention group vs control
- ○ no statistically significant difference between control and intervention
- ○ potentially harmful: control was significantly better than intervention in one or more outcomes
- ○ inconclusive: more research is needed
- ○ Other:

Article Preparation Status/Stage *

At which stage in your article preparation are you currently (at the time you fill in this form)

- ○ not submitted yet - in early draft status
- ○ not submitted yet - in late draft status, just before submission
- ○ submitted to a journal but not reviewed yet
- ○ submitted to a journal and after receiving initial reviewer comments
- ◉ submitted to a journal and accepted, but not published yet
- ○ published
- ○ Other:

Journal *

If you already know where you will submit this paper (or if it is already submitted), please provide the journal name (if it is not JMIR, provide the journal name under "other")

- ( ) not submitted yet / unclear where I will submit this
- (x) Journal of Medical Internet Research (JMIR)
- ( ) JMIR mHealth and UHealth
- ( ) JMIR Serious Games
- ( ) JMIR Mental Health
- ( ) JMIR Public Health
- ( ) JMIR Formative Research
- ( ) Other JMIR sister journal
- ( ) Other:

Is this a full powered effectiveness trial or a pilot/feasibility trial? *

- ( ) Pilot/feasibility
- (x) Fully powered

Manuscript tracking number *

If this is a JMIR submission, please provide the manuscript tracking number under "other" (The ms tracking number can be found in the submission acknowledgement email, or when you login as author in JMIR. If the paper is already published in JMIR, then the ms tracking number is the four-digit number at the end of the DOI, to be found at the bottom of each published article in JMIR)

- ( ) no ms number (yet) / not (yet) submitted to / published in JMIR
- (x) Other: JMIR ms#47266

TITLE AND ABSTRACT

1a) TITLE: Identification as a randomized trial in the title

1a) Does your paper address CONSORT item 1a? *

I.e does the title contain the phrase "Randomized Controlled Trial"? (if not, explain the reason under "other")

- (●) yes
- ( ) Other:

1a-i) Identify the mode of delivery in the title

Identify the mode of delivery. Preferably use “web-based” and/or “mobile” and/or “electronic game” in the title. Avoid ambiguous terms like “online”, “virtual”, “interactive”. Use “Internet-based” only if Intervention includes non-web-based Internet components (e.g. email), use “computer-based” or “electronic” only if offline products are used. Use “virtual” only in the context of “virtual reality” (3-D worlds). Use “online” only in the context of “online support groups”. Complement or substitute product names with broader terms for the class of products (such as “mobile” or “smart phone” instead of “iphone”), especially if the application runs on different platforms.

subitem not at all important

- 1 ( )
- 2 ( )
- 3 (●)
- 4 ( )
- 5 ( )

essential

Clear selection

Does your paper address subitem 1a-i? *

Copy and paste relevant sections from manuscript title (include quotes in quotation marks "like this" to indicate direct quotes from your manuscript), or elaborate on this item by providing additional information not in the ms, or briefly explain why the item is not applicable/relevant for your study

Title: Effect of short, animated video storytelling on maternal knowledge and satisfaction in the perinatal period in South Africa: a randomized controlled trial. Since our animated videos have not been incorporated into an app, we feel the use of the words "animated video" in the title clearly describe what we developed: a set of animated videos.

1a-ii) Non-web-based components or important co-interventions in title

Mention non-web-based components or important co-interventions in title, if any (e.g., “with telephone support”).

subitem not at all important

- 1 (selected)
- 2
- 3
- 4
- 5

essential

Clear selection

Does your paper address subitem 1a-ii?

Copy and paste relevant sections from manuscript title (include quotes in quotation marks "like this" to indicate direct quotes from your manuscript), or elaborate on this item by providing additional information not in the ms, or briefly explain why the item is not applicable/relevant for your study

We did not integrate non-web-based components in this trial.

1a-iii) Primary condition or target group in the title

Mention primary condition or target group in the title, if any (e.g., "for children with Type I Diabetes") Example: A Web-based and Mobile Intervention with Telephone Support for Children with Type I Diabetes: Randomized Controlled Trial

subitem not at all important

1 ○

2 ○

3 ○

4 ○

5 ◉

essential

Clear selection

Does your paper address subitem 1a-iii? *

Copy and paste relevant sections from manuscript title (include quotes in quotation marks "like this" to indicate direct quotes from your manuscript), or elaborate on this item by providing additional information not in the ms, or briefly explain why the item is not applicable/relevant for your study

The title contains the phrase "on maternal knowledge and satisfaction in the perinatal period in South Africa" which elucidates our primary target audience and condition.

1b) ABSTRACT: Structured summary of trial design, methods, results, and conclusions

NPT extension: Description of experimental treatment, comparator, care providers, centers, and blinding status.

1b-i) Key features/functionalities/components of the intervention and comparator in the METHODS section of the ABSTRACT

Mention key features/functionalities/components of the intervention and comparator in the abstract. If possible, also mention theories and principles used for designing the site. Keep in mind the needs of systematic reviewers and indexers by including important synonyms. (Note: Only report in the abstract what the main paper is reporting. If this information is missing from the main body of text, consider adding it)

subitem not at all important

1 ○

2 ○

3 ◉

4 ○

5 ○

essential

Clear selection

Does your paper address subitem 1b-i? *

Copy and paste relevant sections from the manuscript abstract (include quotes in quotation marks "like this" to indicate direct quotes from your manuscript), or elaborate on this item by providing additional information not in the ms, or briefly explain why the item is not applicable/relevant for your study

We discuss the approach we used to developing this video series (animated storytelling), stating in the abstract that "Short, animated storytelling is an innovative, emerging approach to mobile health messaging." AND "While prior studies have shown the promise of animated video health education, most of this research is still being conducted in high-income countries. More research in under-resourced settings is urgently needed, especially as access to mobile technology increases in the Global South."

1b-ii) Level of human involvement in the METHODS section of the ABSTRACT

Clarify the level of human involvement in the abstract, e.g., use phrases like “fully automated” vs. “therapist/nurse/care provider/physician-assisted” (mention number and expertise of providers involved, if any). (Note: Only report in the abstract what the main paper is reporting. If this information is missing from the main body of text, consider adding it)

subitem not at all important

1 ○

2 ○

3 ◉

4 ○

5 ○

essential

Clear selection

Does your paper address subitem 1b-ii?

Copy and paste relevant sections from the manuscript abstract (include quotes in quotation marks "like this" to indicate direct quotes from your manuscript), or elaborate on this item by providing additional information not in the ms, or briefly explain why the item is not applicable/relevant for your study

We state in the abstract that "The intervention videos were delivered via WhatsApp and, one month later, participants responded to telephone surveys assessing knowledge. The intervention group then participated in a nested evaluation of user satisfaction."

1b-iii) Open vs. closed, web-based (self-assessment) vs. face-to-face assessments in the METHODS section of the ABSTRACT

Mention how participants were recruited (online vs. offline), e.g., from an open access website or from a clinic or a closed online user group (closed usergroup trial), and clarify if this was a purely web-based trial, or there were face-to-face components (as part of the intervention or for assessment). Clearly say if outcomes were self-assessed through questionnaires (as common in web-based trials). Note: In traditional offline trials, an open trial (open-label trial) is a type of clinical trial in which both the researchers and participants know which treatment is being administered. To avoid confusion, use “blinded” or “unblinded” to indicated the level of blinding instead of “open”, as “open” in web-based trials usually refers to “open access” (i.e. participants can self-enrol). (Note: Only report in the abstract what the main paper is reporting. If this information is missing from the main body of text, consider adding it)

subitem not at all important

- 1 ○
- 2 ○
- 3 ◉
- 4 ○
- 5 ○

essential

Clear selection

Does your paper address subitem 1b-iii?

Copy and paste relevant sections from the manuscript abstract (include quotes in quotation marks "like this" to indicate direct quotes from your manuscript), or elaborate on this item by providing additional information not in the ms, or briefly explain why the item is not applicable/relevant for your study

In the Abstract we state: "We used a randomized controlled trial with a nested evaluation of user satisfaction to measure the effect of short, animated storytelling (SAS) videos on maternal knowledge and user satisfaction, for mothers enrolled in antenatal care programs at two public health facilities in the Tshwane District of South Africa. Participants were randomized 1:1 into Standard-of-care (SOC) Control and SAS Intervention groups. The intervention videos were delivered via WhatsApp and, one month later, participants responded to telephone surveys assessing knowledge. The intervention group then participated in a nested evaluation of user satisfaction."

1b-iv) RESULTS section in abstract must contain use data

Report number of participants enrolled/assessed in each group, the use/uptake of the intervention (e.g., attrition/adherence metrics, use over time, number of logins etc.), in addition to primary/secondary outcomes. (Note: Only report in the abstract what the main paper is reporting. If this information is missing from the main body of text, consider adding it)

subitem not at all important

1 ○

2 ○

3 ◉

4 ○

5 ○

essential

Clear selection

Does your paper address subitem 1b-iv?

Copy and paste relevant sections from the manuscript abstract (include quotes in quotation marks "like this" to indicate direct quotes from your manuscript), or elaborate on this item by providing additional information not in the ms, or briefly explain why the item is not applicable/relevant for your study

In this trial, we measured the effect on knowledge and user satisfaction of a single instance of viewing a videos series we developed. We make this clear in the abstract. There we no app developed (yet) and the video series has not yet been integrated into any formal URL's. In the Abstract we state: "We surveyed 204 participants. Of these, 49.5% were between the ages of 25 and 34. Almost all participants self-identified as Black African, with the majority (93.2%) having completed secondary school. The mean overall knowledge score was 21.92/28. We observed only a slight increase of 0.28 [95% UI: -0.58–1.16] in the overall knowledge score in the intervention arm. We found that those with secondary education or above, scored higher than those with only primary education, by 2.24 [95% UI: 0.76–4.01]. Participants aged 35+ also scored higher than the youngest age-group 18-24 by 1.83 [95% CI: 0.39–3.33]. Finally, the nested user satisfaction evaluation revealed high maternal satisfaction (4.71/5) with the SAS video series."

1b-v) CONCLUSIONS/DISCUSSION in abstract for negative trials

Conclusions/Discussions in abstract for negative trials: Discuss the primary outcome - if the trial is negative (primary outcome not changed), and the intervention was not used, discuss whether negative results are attributable to lack of uptake and discuss reasons. (Note: Only report in the abstract what the main paper is reporting. If this information is missing from the main body of text, consider adding it)

subitem not at all important

1 ○

2 ○

3 ◉

4 ○

5 ○

essential

Clear selection

Does your paper address subitem 1b-v?

Copy and paste relevant sections from the manuscript abstract (include quotes in quotation marks "like this" to indicate direct quotes from your manuscript), or elaborate on this item by providing additional information not in the ms, or briefly explain why the item is not applicable/relevant for your study

Regarding subitem 1b-v, our Abstract states: "While the SAS videos resulted in high user satisfaction, measured knowledge gains were small within a participant population that was already receiving perinatal health messages through antenatal clinics. The higher knowledge scores observed in older participants with higher education levels suggest that boosting maternal knowledge in younger mothers with lower education levels should continue to be a public health priority in South Africa. While prior studies have shown the promise of animated video health education, most of this research is still being conducted in high-income countries. More research in under-resourced settings is urgently needed, especially as access to mobile technology increases in the Global South. Given the high maternal satisfaction among the SAS video-users in this study, policymakers should consider integrating similar approaches into existing, broad-reaching perinatal health programs, like MomConnect, to boost satisfaction and potentially enhance maternal engagement. Future studies should attempt to quantify the effect of SAS videos on maternal knowledge in hard-to-reach populations who may have limited or no access to antenatal care, although real-world, logistical challenges persist when implementing studies with expectant mothers in under-resourced South African populations."

INTRODUCTION

2a) In INTRODUCTION: Scientific background and explanation of rationale

2a-i) Problem and the type of system/solution

Describe the problem and the type of system/solution that is object of the study: intended as stand-alone intervention vs. incorporated in broader health care program? Intended for a particular patient population? Goals of the intervention, e.g., being more cost-effective to other interventions, replace or complement other solutions? (Note: Details about the intervention are provided in “Methods” under 5)

subitem not at all important

1 ○

2 ○

3 ○

4 ◉

5 ○

essential

Clear selection

Does your paper address subitem 2a-i? *

Copy and paste relevant sections from the manuscript (include quotes in quotation marks "like this" to indicate direct quotes from your manuscript), or elaborate on this item by providing additional information not in the ms, or briefly explain why the item is not applicable/relevant for your study

Regarding subitem 2 a-i, our Introduction states: "Despite documented reductions in maternal and child mortality during the era of the Millennium Development Goals [1-3], millions of women and babies still die each year in South Africa, due to largely preventable perinatal complications [4,5]. An important component of South Africa's efforts to achieve the 2030 Sustainable Development Goals, lies in improving maternal knowledge by increasing access to critical health information in the perinatal period [5,6]. Evidence-based health information and recommendations that motivate life-saving health behaviors often fail to reach mothers because they are too technical or because they fail to engage mothers in ways that optimize user satisfaction during the potentially stressful perinatal period [5,7,8].

Mobile health (mHealth) interventions have shown promise towards broadly disseminating perinatal messages and engaging expectant mothers. As part of their efforts to improve maternal-child health outcomes, the South African National Department of Health introduced an mHealth intervention named "MomConnect" in 2014 [9]. Within 3 years, the program had reached more than 1.5M pregnant women, sending them free, twice-weekly health information text messages [10]. The impressive reach of MomConnect has been documented and supports an increasingly relevant role for mHealth interventions in perinatal care protocols [11].

Text-based dissemination was originally chosen to make MomConnect universally accessible to mothers with basic, non-smart, cell phones [10]. Prior research has documented the broad reach and positive maternal experiences of using the program, suggesting that these could be further enhanced by supporting its accessibility for those who struggle to read or write [12]. In a qualitative study of mothers in rural areas of South Africa, the desire for integrated videos or voice recordings to support health messages [13] also emerged as a theme. The increasing availability of smartphones in South Africa [10,14] presents new opportunities to enhance initiatives like MomConnect, by integrating innovative approaches, like animated video storytelling, to increase accessibility, ease of use, engagement and user satisfaction.

Short, animated storytelling (SAS) videos constitute a promising new approach to public health education that emerged during the COVID-19 pandemic. The development of this approach was catalyzed by the necessity for rapid, accessible global communication of critical health messages [15,16]. The use of visual storytelling greatly increased the accessibility of health messages across languages, education and literacy levels [17]. Subsequent research suggested high engagement and measurable shifts in behavioral intent in response to health messages delivered through narrated videos using a SAS approach [18,19].

Animation has been shown to support patient education [20]. Animated characters also have the potential to resonate across diverse cultural groups [21], like those that make up the rich cultural tapestry of South Africa. Simple, vector animations can also be reduced to small, readily shareable files that can be disseminated on popular platforms like WhatsApp [22]. "Glocalizable" characters, those that can resonate cross-culturally, also facilitate the easy translation of SAS videos into other languages, simply by changing the audio tracks [21].

In this study, named the Amandla Mama study [23], we explore the effect of SAS videos, aligned with MomConnect messaging, on maternal knowledge and maternal satisfaction in the perinatal period. In the South African languages of isiZulu and isiXhosa "Amandla Mama" translates into "Motherly Strength" or "Power to the mother", reflecting the

overarching goals of our intervention.

The specific aims of this study were:
1) to measure the effect of watching the Amandla Mama SAS video series on knowledge of maternal and neonatal health, and
2) to measure user satisfaction associated with the Amandla Mama SAS video series."

2a-ii) Scientific background, rationale: What is known about the (type of) system

Scientific background, rationale: What is known about the (type of) system that is the object of the study (be sure to discuss the use of similar systems for other conditions/diagnoses, if appropiate), motivation for the study, i.e. what are the reasons for and what is the context for this specific study, from which stakeholder viewpoint is the study performed, potential impact of findings [2]. Briefly justify the choice of the comparator.

subitem not at all important

1 ○

2 ○

3 ◉

4 ○

5 ○

essential

Clear selection

Does your paper address subitem 2a-ii? *

Copy and paste relevant sections from the manuscript (include quotes in quotation marks "like this" to indicate direct quotes from your manuscript), or elaborate on this item by providing additional information not in the ms, or briefly explain why the item is not applicable/relevant for your study

Regarding subitem 2a-ii, in the manuscript, we state: "Evidence-based health information and recommendations that motivate life-saving health behaviors often fail to reach mothers because they are too technical or because they fail to engage mothers in ways that optimize user satisfaction during the potentially stressful perinatal period [5,7,8].

Mobile health (mHealth) interventions have shown promise towards broadly disseminating perinatal messages and engaging expectant mothers. As part of their efforts to improve maternal-child health outcomes, the South African National Department of Health introduced an mHealth intervention named “MomConnect” in 2014 [9]. Within 3 years, the program had reached more than 1.5M pregnant women, sending them free, twice-weekly health information text messages [10]. The impressive reach of MomConnect has been documented and supports an increasingly relevant role for mHealth interventions in perinatal care protocols [11].

Text-based dissemination was originally chosen to make MomConnect universally accessible to mothers with basic, non-smart, cell phones [10]. Prior research has documented the broad reach and positive maternal experiences of using the program, suggesting that these could be further enhanced by supporting its accessibility for those who struggle to read or write [12]. In a qualitative study of mothers in rural areas of South Africa, the desire for integrated videos or voice recordings to support health messages [13] also emerged as a theme. The increasing availability of smartphones in South Africa [10,14] presents new opportunities to enhance initiatives like MomConnect, by integrating innovative approaches, like animated video storytelling, to increase accessibility, ease of use, engagement and user satisfaction.

Short, animated storytelling (SAS) videos constitute a promising new approach to public health education that emerged during the COVID-19 pandemic. The development of this approach was catalyzed by the necessity for rapid, accessible global communication of critical health messages [15,16]. The use of visual storytelling greatly increased the accessibility of health messages across languages, education and literacy levels [17]. Subsequent research suggested high engagement and measurable shifts in behavioral intent in response to health messages delivered through narrated videos using a SAS approach [18,19].

Animation has been shown to support patient education [20]. Animated characters also have the potential to resonate across diverse cultural groups [21], like those that make up the rich cultural tapestry of South Africa. Simple, vector animations can also be reduced to small, readily shareable files that can be disseminated on popular platforms like WhatsApp [22]. “Glocalizable” characters, those that can resonate cross-culturally, also facilitate the easy translation of SAS videos into other languages, simply by changing the audio tracks [21]."

2b) In INTRODUCTION: Specific objectives or hypotheses

Does your paper address CONSORT subitem 2b? *

Copy and paste relevant sections from the manuscript (include quotes in quotation marks "like this" to indicate direct quotes from your manuscript), or elaborate on this item by providing additional information not in the ms, or briefly explain why the item is not applicable/relevant for your study

We our specific objectives: "The specific aims of this study were:
1) to measure the effect of watching the Amandla Mama SAS video series on knowledge of maternal and neonatal health, and
2) to measure user satisfaction associated with the Amandla Mama SAS video series."

METHODS

3a) Description of trial design (such as parallel, factorial) including allocation ratio

Does your paper address CONSORT subitem 3a? *

Copy and paste relevant sections from the manuscript (include quotes in quotation marks "like this" to indicate direct quotes from your manuscript), or elaborate on this item by providing additional information not in the ms, or briefly explain why the item is not applicable/relevant for your study

Regarding subitem 3a, our manuscript states:
"We used a randomized controlled trial with a nested user satisfaction evaluation to measure the effect of the Amandla Mama videos on maternal knowledge and to assess maternal satisfaction. Participants were randomly assigned to either the short, animated storytelling videos (SAS) Intervention group or the Standard-of-Care (SOC) Control group. Standard of care in South Africa includes regular antenatal counselling at the local clinic, according to the National Department of Health's Guidelines for Maternity Care in South Africa [25]. This care is free of charge for expectant mothers in South Africa, and it is considered part of primary healthcare. As an adjunct to this maternity care, all pregnant women who register at a maternity clinic in South Africa are offered optional enrolment in MomConnect, a free service which offers informational text messages among other resources for pregnant women. In addition to having access to regular antenatal care, mothers assigned to the SAS Intervention group were sent the 10 SAS intervention videos via WhatsApp."

3b) Important changes to methods after trial commencement (such as eligibility criteria), with reasons

Does your paper address CONSORT subitem 3b? *

Copy and paste relevant sections from the manuscript (include quotes in quotation marks "like this" to indicate direct quotes from your manuscript), or elaborate on this item by providing additional information not in the ms, or briefly explain why the item is not applicable/relevant for your study

We did not change our methods after trial comencement.

3b-i) Bug fixes, Downtimes, Content Changes

Bug fixes, Downtimes, Content Changes: ehealth systems are often dynamic systems. A description of changes to methods therefore also includes important changes made on the intervention or comparator during the trial (e.g., major bug fixes or changes in the functionality or content) (5-iii) and other "unexpected events" that may have influenced study design such as staff changes, system failures/downtimes, etc. [2].

subitem not at all important

- 1 (selected)
- 2
- 3
- 4
- 5

essential

Clear selection

Does your paper address subitem 3b-i?

Copy and paste relevant sections from the manuscript (include quotes in quotation marks "like this" to indicate direct quotes from your manuscript), or elaborate on this item by providing additional information not in the ms, or briefly explain why the item is not applicable/relevant for your study

Since we were not launching an app in this trial, we did not have any bugs or bug fixes, downtimes or content changes.

4a) Eligibility criteria for participants

Does your paper address CONSORT subitem 4a? *

Copy and paste relevant sections from the manuscript (include quotes in quotation marks "like this" to indicate direct quotes from your manuscript), or elaborate on this item by providing additional information not in the ms, or briefly explain why the item is not applicable/relevant for your study

Regarding subitem 4a, our manuscript states: "Eligible participants were expectant mothers accessing routine antenatal healthcare services in the two facilities [23]. A total of 556 pregnant women were approached and screened for eligibility. Of these, 185 were excluded because of barriers that would impact their exposure to the intervention (i.e. lack of reliable access to a cellphone or WhatsApp), leaving 371 who met preliminary eligibility criteria. Of these, 149 participants were excluded when attempts to reach them by phone failed because of invalid or inaccurate telephone numbers, making it impossible for them to take part in the outcomes assessment. Of the 222 enrolled participants who we were able to reach at the 2-week timepoint, 18 opted not to be interviewed due to the following reasons: a) miscarriage, b) newborn had died, c) they were no longer interested, or d) they reported not having watched the videos and therefore did not want to answer questions related to them. Ultimately, we were able to interview 204 participants and our analysis is conducted with that data. Figure 2 summarizes the flow of the trial including the points of exclusion and loss to follow-up."

4a-i) Computer / Internet literacy

Computer / Internet literacy is often an implicit “de facto” eligibility criterion - this should be explicitly clarified.

subitem not at all important

1 (selected)

2

3

4

5

essential

Clear selection

Does your paper address subitem 4a-i?

Copy and paste relevant sections from the manuscript (include quotes in quotation marks "like this" to indicate direct quotes from your manuscript), or elaborate on this item by providing additional information not in the ms, or briefly explain why the item is not applicable/relevant for your study

Since our intervention was not delivered using computers and required no computer or internet literacy, this item does not apply.

4a-ii) Open vs. closed, web-based vs. face-to-face assessments:

Open vs. closed, web-based vs. face-to-face assessments: Mention how participants were recruited (online vs. offline), e.g., from an open access website or from a clinic, and clarify if this was a purely web-based trial, or there were face-to-face components (as part of the intervention or for assessment), i.e., to what degree got the study team to know the participant. In online-only trials, clarify if participants were quasi-anonymous and whether having multiple identities was possible or whether technical or logistical measures (e.g., cookies, email confirmation, phone calls) were used to detect/prevent these.

subitem not at all important

- 1 ○
- 2 ○
- 3 ◉
- 4 ○
- 5 ○

essential

Clear selection

Does your paper address subitem 4a-ii? *

Copy and paste relevant sections from the manuscript (include quotes in quotation marks "like this" to indicate direct quotes from your manuscript), or elaborate on this item by providing additional information not in the ms, or briefly explain why the item is not applicable/relevant for your study

Regarding subitem 4a-ii, this only paritally applies to our study. Our manusciprt states: "Our study took place in the City of Tshwane Metropolitan District of Gauteng, South Africa. The Tshwane Health District is situated in the northern region of Gauteng Province. In 2019, the Tshwane District had a population of more than 3 million people, with a population density of 515/km 2 [26]. The district has seven regions and sixty-eight clinics which serve the seven regions within the district. Addressing high rates of low-birthweight babies by encouraging attendance at antenatal clinics has been a public health priority in the region [26]. Participants in this study were recruited during attendance at two public clinics in the City of Tshwane Metropolitan District. The FF Ribeiro Clinic is an urban community clinic located in the central business district of Tshwane, and the Kgabo Clinic is situated in the large settlement of Winterveld and serves a semi-rural community." AND
"Participants were recruited at the clinics, while they waited for their antenatal appointments. As such, this was a convenience sample, recruited consecutively by research team members affiliated with the Clinton Health Access Initiative in South Africa. . Members of the research team described the trial to mothers and gained informed consent for participation in the study from mothers who expressed an interest in taking part. During recruitment, participants were notified that their participation in the study was voluntary and that they could withdraw at any time without any threat or punitive measures. They were also notified that they would receive airtime vouchers to compensate them for their time and expenses incurred by participating in the study."

4a-iii) Information giving during recruitment

Information given during recruitment. Specify how participants were briefed for recruitment and in the informed consent procedures (e.g., publish the informed consent documentation as appendix, see also item X26), as this information may have an effect on user self-selection, user expectation and may also bias results.

subitem not at all important

1 ○

2 ○

3 ○

4 ◉

5 ○

essential

Clear selection

Does your paper address subitem 4a-iii?

Copy and paste relevant sections from the manuscript (include quotes in quotation marks "like this" to indicate direct quotes from your manuscript), or elaborate on this item by providing additional information not in the ms, or briefly explain why the item is not applicable/relevant for your study

Regarding subitem 4a-iii, our manuscript states: "Participants were recruited at the clinics, while they waited for their antenatal appointments. As such, this was a convenience sample, recruited consecutively by research team members affiliated with the Clinton Health Access Initiative in South Africa. . Members of the research team described the trial to mothers and gained informed consent for participation in the study from mothers who expressed an interest in taking part. During recruitment, participants were notified that their participation in the study was voluntary and that they could withdraw at any time without any threat or punitive measures. They were also notified that they would receive airtime vouchers to compensate them for their time and expenses incurred by participating in the study."

4b) Settings and locations where the data were collected

Does your paper address CONSORT subitem 4b? *

Copy and paste relevant sections from the manuscript (include quotes in quotation marks "like this" to indicate direct quotes from your manuscript), or elaborate on this item by providing additional information not in the ms, or briefly explain why the item is not applicable/relevant for your study

Regarding subitem 4b, our manuscript states: "Our study took place in the City of Tshwane Metropolitan District of Gauteng, South Africa. The Tshwane Health District is situated in the northern region of Gauteng Province. In 2019, the Tshwane District had a population of more than 3 million people, with a population density of 515/km 2 [26]. The district has seven regions and sixty-eight clinics which serve the seven regions within the district. Addressing high rates of low-birthweight babies by encouraging attendance at antenatal clinics has been a public health priority in the region [26]. Participants in this study were recruited during attendance at two public clinics in the City of Tshwane Metropolitan District. The FF Ribeiro Clinic is an urban community clinic located in the central business district of Tshwane, and the Kgabo Clinic is situated in the large settlement of Winterveld and serves a semi-rural community."

4b-i) Report if outcomes were (self-)assessed through online questionnaires

Clearly report if outcomes were (self-)assessed through online questionnaires (as common in web-based trials) or otherwise.

subitem not at all important

- 1 ○
- 2 ○
- 3 ◉
- 4 ○
- 5 ○

essential

Clear selection

Does your paper address subitem 4b-i? *

Copy and paste relevant sections from the manuscript (include quotes in quotation marks "like this" to indicate direct quotes from your manuscript), or elaborate on this item by providing additional information not in the ms, or briefly explain why the item is not applicable/relevant for your study

Our outcomes were not self-assessed but rather data was collected through telephone interviews with participants. The manuscript states: "Sociodemographic data were collected verbally from enrolled participants and immediately entered into a shared spreadsheet by our local research team. Since the trial involved testing and implementing a new intervention, and we found no appropriate perinatal knowledge assessments that had been validated within our study population, the research team designed a survey consisting of 28 knowledge questions based on the intervention (Appendix Table 1). For each knowledge question, mothers could answer “True”, “False” or “I don’t know”. The questions were scored by allocating one point for each correct answer and zero points if participants answered incorrectly or if they answered: “I don’t know.” The overall knowledge score was calculated by summing the total of the individual question scores for the 28 survey questions. Surveys were administered by follow-up telephone calls one month after the participants had been enrolled in the study. The participants in the intervention group were asked additional questions about their experiences with downloading and viewing the SAS video series. In order to quantify user satisfaction, we used Kano’s user experience model [27] to develop a survey measuring maternal satisfaction with the SAS video content. Participants in the intervention group were asked to respond to 8 user experience items, ranking their level of satisfaction on a scale of 1 to 5, where 1 is not at all satisfied and 5 is extremely satisfied. The adapted user satisfaction survey is shown in Table 1."

4b-ii) Report how institutional affiliations are displayed

Report how institutional affiliations are displayed to potential participants [on ehealth media], as affiliations with prestigious hospitals or universities may affect volunteer rates, use, and reactions with regards to an intervention.(Not a required item – describe only if this may bias results)

subitem not at all important

1 (selected)

2

3

4

5

essential

Clear selection

Does your paper address subitem 4b-ii?

Copy and paste relevant sections from the manuscript (include quotes in quotation marks "like this" to indicate direct quotes from your manuscript), or elaborate on this item by providing additional information not in the ms, or briefly explain why the item is not applicable/relevant for your study

We did not display institutional affiliations.

5) The interventions for each group with sufficient details to allow replication, including how and when they were actually administered

5-i) Mention names, credential, affiliations of the developers, sponsors, and owners

Mention names, credential, affiliations of the developers, sponsors, and owners [6] (if authors/evaluators are owners or developer of the software, this needs to be declared in a “Conflict of interest” section or mentioned elsewhere in the manuscript).

subitem not at all important

1 (selected)

2

3

4

5

essential

Clear selection

Does your paper address subitem 5-i?

Copy and paste relevant sections from the manuscript (include quotes in quotation marks "like this" to indicate direct quotes from your manuscript), or elaborate on this item by providing additional information not in the ms, or briefly explain why the item is not applicable/relevant for your study

We did not develop software for this trial - or any form of potentially revenue-generating technology. We just developed a set of story videos and tested these with expectant mothers in South Africa.

5-ii) Describe the history/development process

Describe the history/development process of the application and previous formative evaluations (e.g., focus groups, usability testing), as these will have an impact on adoption/use rates and help with interpreting results.

subitem not at all important

1 ○

2 ○

3 ○

4 ◉

5 ○

essential

Clear selection

Does your paper address subitem 5-ii?

Copy and paste relevant sections from the manuscript (include quotes in quotation marks "like this" to indicate direct quotes from your manuscript), or elaborate on this item by providing additional information not in the ms, or briefly explain why the item is not applicable/relevant for your study

Regarding subitem 5-ii, our manuscript states: "With input from local mothers and community health workers in South Africa, we developed the Amandla Mama intervention - a collection of 10 short, animated storytelling videos focused on critical perinatal health topics. During the content creation process, research partners at the Clinton Health Access Initiative South Africa gathered formative feedback from women attending antenatal clinic visits. These expectant mothers shared their thoughts on a collection of prototypes, originally developed by our co-investigator (MA) and the Digital Medic health education program, based in South Africa and the United States [24]. During the development of the prototypes, we received input from community health workers employed by the DG Murray Trust, a community health organization in South Africa. Using a collaborative, human-centered design process [22], mothers were asked to give feedback on the topics, style, length, narratives, audio and visual design of the prototypes. This feedback was then used to design the final 10 intervention videos, animated by a local South African animator. Prior research on global preferences for animated character design was also used to inform the final intervention videos [21]. The topics covered in the series are described in Figure 1, along with screenshots and the duration of each SAS video. The videos were delivered via WhatsApp to participants in our study and each video could be viewed only once to prevent sharing between mothers in the intervention and control groups."

5-iii) Revisions and updating

Revisions and updating. Clearly mention the date and/or version number of the application/intervention (and comparator, if applicable) evaluated, or describe whether the intervention underwent major changes during the evaluation process, or whether the development and/or content was “frozen” during the trial. Describe dynamic components such as news feeds or changing content which may have an impact on the replicability of the intervention (for unexpected events see item 3b).

subitem not at all important

- 1 (selected)
- 2
- 3
- 4
- 5

essential

Clear selection

Does your paper address subitem 5-iii?

Copy and paste relevant sections from the manuscript (include quotes in quotation marks "like this" to indicate direct quotes from your manuscript), or elaborate on this item by providing additional information not in the ms, or briefly explain why the item is not applicable/relevant for your study

Since we did not develop and app or any kind of software, this does not apply to our trial. There was only one version of the intervention.

5-iv) Quality assurance methods

Provide information on quality assurance methods to ensure accuracy and quality of information provided [1], if applicable.

subitem not at all important

1 ○

2 ○

3 ◉

4 ○

5 ○

essential

Clear selection

Does your paper address subitem 5-iv?

Copy and paste relevant sections from the manuscript (include quotes in quotation marks "like this" to indicate direct quotes from your manuscript), or elaborate on this item by providing additional information not in the ms, or briefly explain why the item is not applicable/relevant for your study

The section in the manuscript detailing the intervention development states: "With input from local mothers and community health workers in South Africa, we developed the Amandla Mama intervention - a collection of 10 short, animated storytelling videos focused on critical perinatal health topics. During the content creation process, research partners at the Clinton Health Access Initiative South Africa gathered formative feedback from women attending antenatal clinic visits. These expectant mothers shared their thoughts on a collection of prototypes, originally developed by our co-investigator (MA) and the Digital Medic health education program, based in South Africa and the United States [24]. During the development of the prototypes, we received input from community health workers employed by the DG Murray Trust, a community health organization in South Africa. Using a collaborative, human-centered design process [22], mothers were asked to give feedback on the topics, style, length, narratives, audio and visual design of the prototypes. This feedback was then used to design the final 10 intervention videos, animated by a local South African animator. Prior research on global preferences for animated character design was also used to inform the final intervention videos [21]. The topics covered in the series are described in Figure 1, along with screenshots and the duration of each SAS video. The videos were delivered via WhatsApp to participants in our study and each video could be viewed only once to prevent sharing between mothers in the intervention and control groups."

5-v) Ensure replicability by publishing the source code, and/or providing screenshots/screen-capture video, and/or providing flowcharts of the algorithms used

Ensure replicability by publishing the source code, and/or providing screenshots/screen-capture video, and/or providing flowcharts of the algorithms used. Replicability (i.e., other researchers should in principle be able to replicate the study) is a hallmark of scientific reporting.

subitem not at all important

1 ○

2 ○

3 ○

4 ◉

5 ○

essential

Clear selection

Does your paper address subitem 5-v?

Copy and paste relevant sections from the manuscript (include quotes in quotation marks "like this" to indicate direct quotes from your manuscript), or elaborate on this item by providing additional information not in the ms, or briefly explain why the item is not applicable/relevant for your study

We have posted all of the intervention videos in the appendix and stated that they are freely available for downloading and use for community health promotion purposes. We did not develop any code. We provide screenshots of the videos in one of the manuscript figures.

5-vi) Digital preservation

Digital preservation: Provide the URL of the application, but as the intervention is likely to change or disappear over the course of the years; also make sure the intervention is archived (Internet Archive, webcitation.org, and/or publishing the source code or screenshots/videos alongside the article). As pages behind login screens cannot be archived, consider creating demo pages which are accessible without login.

subitem not at all important

1 ○

2 ○

3 ○

4 ●

5 ○

essential

Clear selection

Does your paper address subitem 5-vi?

Copy and paste relevant sections from the manuscript (include quotes in quotation marks "like this" to indicate direct quotes from your manuscript), or elaborate on this item by providing additional information not in the ms, or briefly explain why the item is not applicable/relevant for your study

The videos are accessible through the manuscript appendix and via a Google Drive folder that will remain open access in perpetuity.

5-vii) Access

Access: Describe how participants accessed the application, in what setting/context, if they had to pay (or were paid) or not, whether they had to be a member of specific group. If known, describe how participants obtained “access to the platform and Internet” [1]. To ensure access for editors/reviewers/readers, consider to provide a “backdoor” login account or demo mode for reviewers/readers to explore the application (also important for archiving purposes, see vi).

subitem not at all important

1 ○

2 ○

3 ○

4 ◉

5 ○

essential

Clear selection

Does your paper address subitem 5-vii? *

Copy and paste relevant sections from the manuscript (include quotes in quotation marks "like this" to indicate direct quotes from your manuscript), or elaborate on this item by providing additional information not in the ms, or briefly explain why the item is not applicable/relevant for your study

In the appendix, we state:
Multimedia Appendix
"The entire video series can be accessed freely.[28] Please contact the first author if you would like to reuse any of these videos for your health promotion work. We would be delighted to share them with you for that purpose."

5-viii) Mode of delivery, features/functionalities/components of the intervention and comparator, and the theoretical framework

Describe mode of delivery, features/functionalities/components of the intervention and comparator, and the theoretical framework [6] used to design them (instructional strategy [1], behaviour change techniques, persuasive features, etc., see e.g., [7, 8] for terminology). This includes an in-depth description of the content (including where it is coming from and who developed it) [1],” whether [and how] it is tailored to individual circumstances and allows users to track their progress and receive feedback” [6]. This also includes a description of communication delivery channels and – if computer-mediated communication is a component – whether communication was synchronous or asynchronous [6]. It also includes information on presentation strategies [1], including page design principles, average amount of text on pages, presence of hyperlinks to other resources, etc. [1].

subitem not at all important

1 ○

2 ○

3 ◉

4 ○

5 ○

essential

Clear selection

Does your paper address subitem 5-viii? *

Copy and paste relevant sections from the manuscript (include quotes in quotation marks "like this" to indicate direct quotes from your manuscript), or elaborate on this item by providing additional information not in the ms, or briefly explain why the item is not applicable/relevant for your study

We describe the development and delivery of the videos in the methods section: "With input from local mothers and community health workers in South Africa, we developed the Amandla Mama intervention - a collection of 10 short, animated storytelling videos focused on critical perinatal health topics. During the content creation process, research partners at the Clinton Health Access Initiative South Africa gathered formative feedback from women attending antenatal clinic visits. These expectant mothers shared their thoughts on a collection of prototypes, originally developed by our co-investigator (MA) and the Digital Medic health education program, based in South Africa and the United States [24]. During the development of the prototypes, we received input from community health workers employed by the DG Murray Trust, a community health organization in South Africa. Using a collaborative, human-centered design process [22], mothers were asked to give feedback on the topics, style, length, narratives, audio and visual design of the prototypes. This feedback was then used to design the final 10 intervention videos, animated by a local South African animator. Prior research on global preferences for animated character design was also used to inform the final intervention videos [21]. The topics covered in the series are described in Figure 1, along with screenshots and the duration of each SAS video. The videos were delivered via WhatsApp to participants in our study and each video could be viewed only once to prevent sharing between mothers in the intervention and control groups." AND "Upon enrolling in the study, participants in the intervention group received the video intervention via WhatsApp, delivered to their cellphones. One month later, all of the participants were contacted telephonically by the research team to respond to the surveys. Following unblinding of the interviewers, participants in the intervention group were asked if they had watched the videos after receiving them and the participants in the control group were asked if they had been shown any short, animated maternal health videos on the phones of others. These questions allowed us to detect any contamination of the control group and also facilitated an analysis based on intention to treat."

5-ix) Describe use parameters

Describe use parameters (e.g., intended “doses” and optimal timing for use). Clarify what instructions or recommendations were given to the user, e.g., regarding timing, frequency, heaviness of use, if any, or was the intervention used ad libitum.

subitem not at all important

- 1 (selected)
- 2
- 3
- 4
- 5

essential

Clear selection

Does your paper address subitem 5-ix?

Copy and paste relevant sections from the manuscript (include quotes in quotation marks "like this" to indicate direct quotes from your manuscript), or elaborate on this item by providing additional information not in the ms, or briefly explain why the item is not applicable/relevant for your study

This study involved the participants receiving short animated videos via whatsapp. The participants in the intervention arm viewed the videos only once. This is described in detail in the methods section: "Upon enrolling in the study, participants in the intervention group received the video intervention via WhatsApp, delivered to their cellphones. One month later, all of the participants were contacted telephonically by the research team to respond to the surveys. Following unblinding of the interviewers, participants in the intervention group were asked if they had watched the videos after receiving them and the participants in the control group were asked if they had been shown any short, animated maternal health videos on the phones of others. These questions allowed us to detect any contamination of the control group and also facilitated an analysis based on intention to treat."

5-x) Clarify the level of human involvement

Clarify the level of human involvement (care providers or health professionals, also technical assistance) in the e-intervention or as co-intervention (detail number and expertise of professionals involved, if any, as well as “type of assistance offered, the timing and frequency of the support, how it is initiated, and the medium by which the assistance is delivered”. It may be necessary to distinguish between the level of human involvement required for the trial, and the level of human involvement required for a routine application outside of a RCT setting (discuss under item 21 – generalizability).

subitem not at all important

1 (selected)

2

3

4

5

essential

Clear selection

Does your paper address subitem 5-x?

Copy and paste relevant sections from the manuscript (include quotes in quotation marks "like this" to indicate direct quotes from your manuscript), or elaborate on this item by providing additional information not in the ms, or briefly explain why the item is not applicable/relevant for your study

In our study, participants were recruited in person during their perinatal visits and they were then interviewed by research assistants after receiving the intervention: "Participants in this study were recruited during attendance at two public clinics in the City of Tshwane Metropolitan District. The FF Ribeiro Clinic is an urban community clinic located in the central business district of Tshwane, and the Kgabo Clinic is situated in the large settlement of Winterveld and serves a semi-rural community.

Recruitment and informed consent
Participants were recruited at the clinics, while they waited for their antenatal appointments. As such, this was a convenience sample, recruited consecutively by research team members affiliated with the Clinton Health Access Initiative in South Africa. . Members of the research team described the trial to mothers and gained informed consent for participation in the study from mothers who expressed an interest in taking part. During recruitment, participants were notified that their participation in the study was voluntary and that they could withdraw at any time without any threat or punitive measures. They were also notified that they would receive airtime vouchers to compensate them for their time and expenses incurred by participating in the study." AND
"Sociodemographic data were collected verbally from enrolled participants and immediately entered into a shared spreadsheet by our local research team. Since the trial involved testing and implementing a new intervention, and we found no appropriate perinatal knowledge assessments that had been validated within our study population, the research team designed a survey consisting of 28 knowledge questions based on the intervention (Appendix Table 1). For each knowledge question, mothers could answer “True”, “False” or “I don’t know”. The questions were scored by allocating one point for each correct answer and zero points if participants answered incorrectly or if they answered: “I don’t know.” The overall knowledge score was calculated by summing the total of the individual question scores for the 28 survey questions. Surveys were administered by follow-up telephone calls one month after the participants had been enrolled in the study. The participants in the intervention group were asked additional questions about their experiences with downloading and viewing the SAS video series. In order to quantify user satisfaction, we used Kano’s user experience model [27] to develop a survey measuring maternal satisfaction with the SAS video content. Participants in the intervention group were asked to respond to 8 user experience items, ranking their level of satisfaction on a scale of 1 to 5, where 1 is not at all satisfied and 5 is extremely satisfied. The adapted user satisfaction survey is shown in Table 1."

5-xi) Report any prompts/reminders used

Report any prompts/reminders used: Clarify if there were prompts (letters, emails, phone calls, SMS) to use the application, what triggered them, frequency etc. It may be necessary to distinguish between the level of prompts/reminders required for the trial, and the level of prompts/reminders for a routine application outside of a RCT setting (discuss under item 21 – generalizability).

subitem not at all important

1 (selected)

2

3

4

5

essential

Clear selection

Does your paper address subitem 5-xi? *

Copy and paste relevant sections from the manuscript (include quotes in quotation marks "like this" to indicate direct quotes from your manuscript), or elaborate on this item by providing additional information not in the ms, or briefly explain why the item is not applicable/relevant for your study

No prompts or reminders were used.

5-xii) Describe any co-interventions (incl. training/support)

Describe any co-interventions (incl. training/support): Clearly state any interventions that are provided in addition to the targeted eHealth intervention, as ehealth intervention may not be designed as stand-alone intervention. This includes training sessions and support [1]. It may be necessary to distinguish between the level of training required for the trial, and the level of training for a routine application outside of a RCT setting (discuss under item 21 – generalizability.

subitem not at all important

- 1 (selected)
- 2
- 3
- 4
- 5

essential

Clear selection

Does your paper address subitem 5-xii? *

Copy and paste relevant sections from the manuscript (include quotes in quotation marks "like this" to indicate direct quotes from your manuscript), or elaborate on this item by providing additional information not in the ms, or briefly explain why the item is not applicable/relevant for your study

No co-interventions were used.

6a) Completely defined pre-specified primary and secondary outcome measures, including how and when they were assessed

Does your paper address CONSORT subitem 6a? *

Copy and paste relevant sections from the manuscript (include quotes in quotation marks "like this" to indicate direct quotes from your manuscript), or elaborate on this item by providing additional information not in the ms, or briefly explain why the item is not applicable/relevant for your study

Our primary and secondary outcome measures were pre-specified and registered. We state: "Outcomes
The primary outcome in this study was maternal knowledge. The secondary outcome was maternal satisfaction, defined as the mean response of all participants in the SAS Intervention group for each of the 8 satisfaction items."
AND "Trial registration
This trial was registered on 14 March 2022 with the Pan African Clinical Trials Registry (PACTR), registration number PACTR202203673222680."

6a-i) Online questionnaires: describe if they were validated for online use and apply CHERRIES items to describe how the questionnaires were designed/deployed

If outcomes were obtained through online questionnaires, describe if they were validated for online use and apply CHERRIES items to describe how the questionnaires were designed/deployed [9].

subitem not at all important

1 ○

2 ○

3 ◉

4 ○

5 ○

essential

Clear selection

Does your paper address subitem 6a-i?

Copy and paste relevant sections from manuscript text

We describe the measures used: "Sociodemographic data were collected verbally from enrolled participants and immediately entered into a shared spreadsheet by our local research team. Since the trial involved testing and implementing a new intervention, and we found no appropriate perinatal knowledge assessments that had been validated within our study population, the research team designed a survey consisting of 28 knowledge questions based on the intervention (Appendix Table 1). For each knowledge question, mothers could answer “True”, “False” or “I don’t know”. The questions were scored by allocating one point for each correct answer and zero points if participants answered incorrectly or if they answered: “I don’t know.” The overall knowledge score was calculated by summing the total of the individual question scores for the 28 survey questions. Surveys were administered by follow-up telephone calls one month after the participants had been enrolled in the study. The participants in the intervention group were asked additional questions about their experiences with downloading and viewing the SAS video series. In order to quantify user satisfaction, we used Kano’s user experience model [27] to develop a survey measuring maternal satisfaction with the SAS video content. Participants in the intervention group were asked to respond to 8 user experience items, ranking their level of satisfaction on a scale of 1 to 5, where 1 is not at all satisfied and 5 is extremely satisfied. The adapted user satisfaction survey is shown in Table 1."

6a-ii) Describe whether and how “use” (including intensity of use/dosage) was defined/measured/monitored

Describe whether and how “use” (including intensity of use/dosage) was defined/measured/monitored (logins, logfile analysis, etc.). Use/adoption metrics are important process outcomes that should be reported in any ehealth trial.

subitem not at all important

- 1 (selected)
- 2
- 3
- 4
- 5

essential

Clear selection

Does your paper address subitem 6a-ii?

Copy and paste relevant sections from manuscript text

Participants watched the videos once. We state this clearly in the manuscript.

6a-iii) Describe whether, how, and when qualitative feedback from participants was obtained

Describe whether, how, and when qualitative feedback from participants was obtained (e.g., through emails, feedback forms, interviews, focus groups).

subitem not at all important

1 ○

2 ○

3 ○

4 ●

5 ○

essential

Clear selection

Does your paper address subitem 6a-iii?

Copy and paste relevant sections from manuscript text

We state that the maternal satisfaction measure was used to solicit feedback from participants: "Participants in the intervention group were asked to respond to 8 user experience items, ranking their level of satisfaction on a scale of 1 to 5, where 1 is not at all satisfied and 5 is extremely satisfied. The adapted user satisfaction survey is shown in Table 1."

6b) Any changes to trial outcomes after the trial commenced, with reasons

Does your paper address CONSORT subitem 6b? *

Copy and paste relevant sections from the manuscript (include quotes in quotation marks "like this" to indicate direct quotes from your manuscript), or elaborate on this item by providing additional information not in the ms, or briefly explain why the item is not applicable/relevant for your study

No changes.

Your answer must have a minimum of 25 characters.

7a) How sample size was determined

NPT: When applicable, details of whether and how the clustering by care provides or centers was addressed

7a-i) Describe whether and how expected attrition was taken into account when calculating the sample size

Describe whether and how expected attrition was taken into account when calculating the sample size.

subitem not at all important

1 ○

2 ○

3 ○

4 ◉

5 ○

essential

Clear selection

Does your paper address subitem 7a-i?

Copy and paste relevant sections from manuscript title (include quotes in quotation marks "like this" to indicate direct quotes from your manuscript), or elaborate on this item by providing additional information not in the ms, or briefly explain why the item is not applicable/relevant for your study

We describe the sample size calculation in the methods section: "We determined the sample size for this trial based on our primary outcome, maternal knowledge. We used a significance level of 0.05 (P<.05) and a power of 0.9, with a two-tailed test for significance. We hypothesized that, at the two-week data collection timepoint, the mean score of participants in the control group on the knowledge questionnaire would be 43% (12 points out of 28 points in the survey), while the mean score of the intervention group on this questionnaire would be 61% (17 out of 28 points). This would constitute an 18% absolute increase and a 1.41 times relative increase. We calculated a sample size of n=140 for each study arm, then added 50% to account for participant drop-outs and loss to follow-up. This brought our sample size to n=210 for the intervention group and n=210 for the control group. Thus, we estimated a total sample size of N=420 participants for the trial. We carried out these sample size calculations on G-Power 3.1.9.6, based on the difference between two independent proportions, using a z-test (Power: 0.9, Alpha 0.05, Allocation n1/n2: 1, p1=0.43, p2=0.61). Finally, we rounded up the sample size per group to the nearest 10 patients (n1=140, n2=140) [23]."

7b) When applicable, explanation of any interim analyses and stopping guidelines

Does your paper address CONSORT subitem 7b? *

Copy and paste relevant sections from the manuscript (include quotes in quotation marks "like this" to indicate direct quotes from your manuscript), or elaborate on this item by providing additional information not in the ms, or briefly explain why the item is not applicable/relevant for your study

We conducted no interim analyses.

8a) Method used to generate the random allocation sequence

NPT: When applicable, how care providers were allocated to each trial group

Does your paper address CONSORT subitem 8a? *

Copy and paste relevant sections from the manuscript (include quotes in quotation marks "like this" to indicate direct quotes from your manuscript), or elaborate on this item by providing additional information not in the ms, or briefly explain why the item is not applicable/relevant for your study

This is described in the manuscript: "Participants were randomly assigned 1:1 to either the SAS Intervention group or the SOC Control group. A computer-generated randomization sequence was used to assign participants to their respective groups. To implement the randomization, a table with the randomized sequence was created and participants were added sequentially as they were enrolled in the study. The randomization sequence was masked and applied to the table by a member of the research team who was not involved in recruiting or interviewing participants. In this way, participants were allocated equally to either the control or intervention group, without the recruiters or the interviewers being aware of the group to which individual participants had been assigned. In this way, both the recruiters and interviewers remained blinded to the allocation throughout the study. After the knowledge survey had been administered, the interviewers were unblinded and able to administer the maternal satisfaction questionnaire, focused on experiences using the videos, to the SAS Intervention mothers."

8b) Type of randomisation; details of any restriction (such as blocking and block size)

Does your paper address CONSORT subitem 8b? *

Copy and paste relevant sections from the manuscript (include quotes in quotation marks "like this" to indicate direct quotes from your manuscript), or elaborate on this item by providing additional information not in the ms, or briefly explain why the item is not applicable/relevant for your study

See above, included in item 8a

9) Mechanism used to implement the random allocation sequence (such as sequentially numbered containers), describing any steps taken to conceal the sequence until interventions were assigned

Does your paper address CONSORT subitem 9? *

Copy and paste relevant sections from the manuscript (include quotes in quotation marks "like this" to indicate direct quotes from your manuscript), or elaborate on this item by providing additional information not in the ms, or briefly explain why the item is not applicable/relevant for your study

See above, included in item 8a

10) Who generated the random allocation sequence, who enrolled participants, and who assigned participants to interventions

Does your paper address CONSORT subitem 10? *

Copy and paste relevant sections from the manuscript (include quotes in quotation marks "like this" to indicate direct quotes from your manuscript), or elaborate on this item by providing additional information not in the ms, or briefly explain why the item is not applicable/relevant for your study

From the manuscript: "Participants were randomly assigned 1:1 to either the SAS Intervention group or the SOC Control group. A computer-generated randomization sequence was used to assign participants to their respective groups. To implement the randomization, a table with the randomized sequence was created and participants were added sequentially as they were enrolled in the study. The randomization sequence was masked and applied to the table by a member of the research team who was not involved in recruiting or interviewing participants. In this way, participants were allocated equally to either the control or intervention group, without the recruiters or the interviewers being aware of the group to which individual participants had been assigned. In this way, both the recruiters and interviewers remained blinded to the allocation throughout the study. After the knowledge survey had been administered, the interviewers were unblinded and able to administer the maternal satisfaction questionnaire, focused on experiences using the videos, to the SAS Intervention mothers."

11a) If done, who was blinded after assignment to interventions (for example, participants, care providers, those assessing outcomes) and how

NPT: Whether or not administering co-interventions were blinded to group assignment

11a-i) Specify who was blinded, and who wasn't

Specify who was blinded, and who wasn't. Usually, in web-based trials it is not possible to blind the participants [1, 3] (this should be clearly acknowledged), but it may be possible to blind outcome assessors, those doing data analysis or those administering co-interventions (if any).

subitem not at all important

1 ○

2 ○

3 ◉

4 ○

5 ○

essential

Clear selection

Does your paper address subitem 11a-i? *

Copy and paste relevant sections from the manuscript (include quotes in quotation marks "like this" to indicate direct quotes from your manuscript), or elaborate on this item by providing additional information not in the ms, or briefly explain why the item is not applicable/relevant for your study

From the manuscript: "The randomization sequence was masked and applied to the table by a member of the research team who was not involved in recruiting or interviewing participants. In this way, participants were allocated equally to either the control or intervention group, without the recruiters or the interviewers being aware of the group to which individual participants had been assigned. In this way, both the recruiters and interviewers remained blinded to the allocation throughout the study. After the knowledge survey had been administered, the interviewers were unblinded and able to administer the maternal satisfaction questionnaire, focused on experiences using the videos, to the SAS Intervention mothers."

11a-ii) Discuss e.g., whether participants knew which intervention was the “intervention of interest” and which one was the “comparator”

Informed consent procedures (4a-ii) can create biases and certain expectations - discuss e.g., whether participants knew which intervention was the “intervention of interest” and which one was the “comparator”.

subitem not at all important

- (x) 1
- ( ) 2
- ( ) 3
- ( ) 4
- ( ) 5

essential

Clear selection

Does your paper address subitem 11a-ii?

Copy and paste relevant sections from the manuscript (include quotes in quotation marks "like this" to indicate direct quotes from your manuscript), or elaborate on this item by providing additional information not in the ms, or briefly explain why the item is not applicable/relevant for your study

See item 11-a-i for this description.

11b) If relevant, description of the similarity of interventions

(this item is usually not relevant for ehealth trials as it refers to similarity of a placebo or sham intervention to a active medication/intervention)

Does your paper address CONSORT subitem 11b? *

Copy and paste relevant sections from the manuscript (include quotes in quotation marks "like this" to indicate direct quotes from your manuscript), or elaborate on this item by providing additional information not in the ms, or briefly explain why the item is not applicable/relevant for your study

This is not applicable in our study.

12a) Statistical methods used to compare groups for primary and secondary outcomes

NPT: When applicable, details of whether and how the clustering by care providers or centers was addressed

Does your paper address CONSORT subitem 12a? *

Copy and paste relevant sections from the manuscript (include quotes in quotation marks "like this" to indicate direct quotes from your manuscript), or elaborate on this item by providing additional information not in the ms, or briefly explain why the item is not applicable/relevant for your study

We include this in the manuscript: "

12a-i) Imputation techniques to deal with attrition / missing values

Imputation techniques to deal with attrition / missing values: Not all participants will use the intervention/comparator as intended and attrition is typically high in ehealth trials. Specify how participants who did not use the application or dropped out from the trial were treated in the statistical analysis (a complete case analysis is strongly discouraged, and simple imputation techniques such as LOCF may also be problematic [4]).

subitem not at all important

1 (selected)

2

3

4

5

essential

Clear selection

Does your paper address subitem 12a-i? *

Copy and paste relevant sections from the manuscript (include quotes in quotation marks "like this" to indicate direct quotes from your manuscript), or elaborate on this item by providing additional information not in the ms, or briefly explain why the item is not applicable/relevant for your study

This is not applicable in our case.

12b) Methods for additional analyses, such as subgroup analyses and adjusted analyses

Does your paper address CONSORT subitem 12b? *

Copy and paste relevant sections from the manuscript (include quotes in quotation marks "like this" to indicate direct quotes from your manuscript), or elaborate on this item by providing additional information not in the ms, or briefly explain why the item is not applicable/relevant for your study

We state in the manuscript: "We compared the distribution of demographic variables between the trial arms with Chi-square or the exact Fisher test as appropriate. Regarding the primary outcome, the sum of the score is a discrete count, but with a bounded value at the total number of questions (28 knowledge questions). Thus, we modelled the total score using a Beta-Binomial distribution, i.e., score~BB(n=28,μ,σ), which accounts for potential over-dispersion and the bounded property of the distribution. Compared to the empirical distribution of the total score, the distribution fitted better than the common count distributions, including Poisson and Negative Binomial (Appendix – Figure 1).

There were 11 individuals with missing data in one of the 28 knowledge questions; thus, we used multiple imputations to impute the missing values and conducted analyses on 100 imputed samples and reported the pooled analyses. We conducted multivariable regression analyses to explore the effect on the knowledge scores of the SAS Intervention group based on intention to treat. The model was adjusted for age, sex, education level, living area, working status, as well as whether the mother enrolled in the MomConnect program. The models were fitted in a Bayesian inference framework using the statistical program R 4.0.2. We reported the 95% uncertainty interval of the mean differences between the covariates by drawing 1000 samples from the posterior distribution and calculating the quantiles of the mean difference, which is nμ for the BB distribution. (The code for analyses is publicly available at https://github.com/kklot/mama). We analyzed the maternal satisfaction data by calculating a mean score for each of the different subjective user satisfaction questions, to capture maternal satisfaction related to the use of the SAS videos. Finally, we measured the internal reliability of the maternal satisfaction items using a Cronbach Alpha test."

X26) REB/IRB Approval and Ethical Considerations [recommended as subheading under "Methods"] (not a CONSORT item)

X26-i) Comment on ethics committee approval

subitem not at all important

1 ○

2 ○

3 ○

4 ○

5 ◉

essential

Clear selection

Does your paper address subitem X26-i?

Copy and paste relevant sections from the manuscript (include quotes in quotation marks "like this" to indicate direct quotes from your manuscript), or elaborate on this item by providing additional information not in the ms, or briefly explain why the item is not applicable/relevant for your study

Yes. We state: "Ethical approval for the study was granted by the Pharma-Ethics Independent Research Ethics Committee, the Tshwane Health Research and Ethics Committee and the Scientific Research Ethics Committee of the Clinton Health Access Initiative. We also registered our study on the National Health Research Database (ref:GP202111071). The study researchers were trained in Good Clinical Practice through accredited health research ethics training programs. Written informed consent was obtained from all participants."

x26-ii) Outline informed consent procedures

Outline informed consent procedures e.g., if consent was obtained offline or online (how? Checkbox, etc.?), and what information was provided (see 4a-ii). See [6] for some items to be included in informed consent documents.

subitem not at all important

1 ○

2 ○

3 ○

4 ○

5 ◉

essential

Clear selection

Does your paper address subitem X26-ii?

Copy and paste relevant sections from the manuscript (include quotes in quotation marks "like this" to indicate direct quotes from your manuscript), or elaborate on this item by providing additional information not in the ms, or briefly explain why the item is not applicable/relevant for your study

Yes this is detailed extensively in the sections quoted above and repeated here: "Written informed consent was obtained from all participants."

X26-iii) Safety and security procedures

Safety and security procedures, incl. privacy considerations, and any steps taken to reduce the likelihood or detection of harm (e.g., education and training, availability of a hotline)

subitem not at all important

1 (selected)

2

3

4

5

essential

Clear selection

Does your paper address subitem X26-iii?

Copy and paste relevant sections from the manuscript (include quotes in quotation marks "like this" to indicate direct quotes from your manuscript), or elaborate on this item by providing additional information not in the ms, or briefly explain why the item is not applicable/relevant for your study

Since this was a trial of short, animated educational videos, the intervention was deemed to be extremely low risk. We did make sure to protect the anonymity and privacy of all participants.

RESULTS

13a) For each group, the numbers of participants who were randomly assigned, received intended treatment, and were analysed for the primary outcome

NPT: The number of care providers or centers performing the intervention in each group and the number of patients treated by each care provider in each center

Does your paper address CONSORT subitem 13a? *

Copy and paste relevant sections from the manuscript (include quotes in quotation marks "like this" to indicate direct quotes from your manuscript), or elaborate on this item by providing additional information not in the ms, or briefly explain why the item is not applicable/relevant for your study

We state this in Figure 2.

13b) For each group, losses and exclusions after randomisation, together with reasons

Does your paper address CONSORT subitem 13b? (NOTE: Preferably, this is shown in a CONSORT flow diagram) *

Copy and paste relevant sections from the manuscript (include quotes in quotation marks "like this" to indicate direct quotes from your manuscript), or elaborate on this item by providing additional information not in the ms, or briefly explain why the item is not applicable/relevant for your study

This is shown in Figure 2.

13b-i) Attrition diagram

Strongly recommended: An attrition diagram (e.g., proportion of participants still logging in or using the intervention/comparator in each group plotted over time, similar to a survival curve) or other figures or tables demonstrating usage/dose/engagement.

subitem not at all important

1 ○

2 ○

3 ○

4 ○

5 ◉

essential

Clear selection

Does your paper address subitem 13b-i?

Copy and paste relevant sections from the manuscript or cite the figure number if applicable (include quotes in quotation marks "like this" to indicate direct quotes from your manuscript), or elaborate on this item by providing additional information not in the ms, or briefly explain why the item is not applicable/relevant for your study

This is shown in Figure 2.

14a) Dates defining the periods of recruitment and follow-up

Does your paper address CONSORT subitem 14a? *

Copy and paste relevant sections from the manuscript (include quotes in quotation marks "like this" to indicate direct quotes from your manuscript), or elaborate on this item by providing additional information not in the ms, or briefly explain why the item is not applicable/relevant for your study

Yes we specify dates in the manuscript in the Participant Timeline within the Methods section.

14a-i) Indicate if critical “secular events” fell into the study period

Indicate if critical “secular events” fell into the study period, e.g., significant changes in Internet resources available or “changes in computer hardware or Internet delivery resources”

subitem not at all important

1 (selected)

2

3

4

5

essential

Clear selection

Does your paper address subitem 14a-i?

Copy and paste relevant sections from the manuscript (include quotes in quotation marks "like this" to indicate direct quotes from your manuscript), or elaborate on this item by providing additional information not in the ms, or briefly explain why the item is not applicable/relevant for your study

This is not applicable for our study.

14b) Why the trial ended or was stopped (early)

Does your paper address CONSORT subitem 14b? *

Copy and paste relevant sections from the manuscript (include quotes in quotation marks "like this" to indicate direct quotes from your manuscript), or elaborate on this item by providing additional information not in the ms, or briefly explain why the item is not applicable/relevant for your study

Our trial was not stopped early.

15) A table showing baseline demographic and clinical characteristics for each group

NPT: When applicable, a description of care providers (case volume, qualification, expertise, etc.) and centers (volume) in each group

Does your paper address CONSORT subitem 15? *

Copy and paste relevant sections from the manuscript (include quotes in quotation marks "like this" to indicate direct quotes from your manuscript), or elaborate on this item by providing additional information not in the ms, or briefly explain why the item is not applicable/relevant for your study

We show baseline demographic data in Table 2.

15-i) Report demographics associated with digital divide issues

In ehealth trials it is particularly important to report demographics associated with digital divide issues, such as age, education, gender, social-economic status, computer/Internet/ehealth literacy of the participants, if known.

subitem not at all important

1 ○

2 ○

3 ○

4 ○

5 ◉

essential

Clear selection

Does your paper address subitem 15-i? *

Copy and paste relevant sections from the manuscript (include quotes in quotation marks "like this" to indicate direct quotes from your manuscript), or elaborate on this item by providing additional information not in the ms, or briefly explain why the item is not applicable/relevant for your study

The purpose of this study was to make video-based health education available to an underserved South African population. Bridging the digital divide is a major goal of the study.

16) For each group, number of participants (denominator) included in each analysis and whether the analysis was by original assigned groups

16-i) Report multiple “denominators” and provide definitions

Report multiple “denominators” and provide definitions: Report N’s (and effect sizes) “across a range of study participation [and use] thresholds” [1], e.g., N exposed, N consented, N used more than x times, N used more than y weeks, N participants “used” the intervention/comparator at specific pre-defined time points of interest (in absolute and relative numbers per group). Always clearly define “use” of the intervention.

subitem not at all important

1 ○

2 ○

3 ○

4 ○

5 ◉

essential

Clear selection

Does your paper address subitem 16-i? *

Copy and paste relevant sections from the manuscript (include quotes in quotation marks "like this" to indicate direct quotes from your manuscript), or elaborate on this item by providing additional information not in the ms, or briefly explain why the item is not applicable/relevant for your study

Yes. We have provided denominators throughout the manuscript.

16-ii) Primary analysis should be intent-to-treat

Primary analysis should be intent-to-treat, secondary analyses could include comparing only "users", with the appropriate caveats that this is no longer a randomized sample (see 18-i).

subitem not at all important

1 ○

2 ○

3 ○

4 ○

5 ◉

essential

Clear selection

Does your paper address subitem 16-ii?

Copy and paste relevant sections from the manuscript (include quotes in quotation marks "like this" to indicate direct quotes from your manuscript), or elaborate on this item by providing additional information not in the ms, or briefly explain why the item is not applicable/relevant for your study

"We conducted multivariable regression analyses to explore the effect on the knowledge scores of the SAS Intervention group based on intention to treat."

17a) For each primary and secondary outcome, results for each group, and the estimated effect size and its precision (such as 95% confidence interval)

Does your paper address CONSORT subitem 17a? *

Copy and paste relevant sections from the manuscript (include quotes in quotation marks "like this" to indicate direct quotes from your manuscript), or elaborate on this item by providing additional information not in the ms, or briefly explain why the item is not applicable/relevant for your study

We present confidence intervals throughout when presenting results.

17a-i) Presentation of process outcomes such as metrics of use and intensity of use

In addition to primary/secondary (clinical) outcomes, the presentation of process outcomes such as metrics of use and intensity of use (dose, exposure) and their operational definitions is critical. This does not only refer to metrics of attrition (13-b) (often a binary variable), but also to more continuous exposure metrics such as "average session length". These must be accompanied by a technical description how a metric like a "session" is defined (e.g., timeout after idle time) [1] (report under item 6a).

subitem not at all important

- 1 (selected)
- 2
- 3
- 4
- 5

essential

Clear selection

Does your paper address subitem 17a-i?

Copy and paste relevant sections from the manuscript (include quotes in quotation marks "like this" to indicate direct quotes from your manuscript), or elaborate on this item by providing additional information not in the ms, or briefly explain why the item is not applicable/relevant for your study

Since our study is not evaluating an app, rather the effect of a one-time exposure to a novel intervention, we are not able to track metrics.

17b) For binary outcomes, presentation of both absolute and relative effect sizes is recommended

Does your paper address CONSORT subitem 17b? *

Copy and paste relevant sections from the manuscript (include quotes in quotation marks "like this" to indicate direct quotes from your manuscript), or elaborate on this item by providing additional information not in the ms, or briefly explain why the item is not applicable/relevant for your study

In our Results section, we present both absolute and relative effect sizes.

18) Results of any other analyses performed, including subgroup analyses and adjusted analyses, distinguishing pre-specified from exploratory

Does your paper address CONSORT subitem 18? *

Copy and paste relevant sections from the manuscript (include quotes in quotation marks "like this" to indicate direct quotes from your manuscript), or elaborate on this item by providing additional information not in the ms, or briefly explain why the item is not applicable/relevant for your study

We present all results in the results section, including subgroup analyses. These are described in detail in the Results section.

18-i) Subgroup analysis of comparing only users

A subgroup analysis of comparing only users is not uncommon in ehealth trials, but if done, it must be stressed that this is a self-selected sample and no longer an unbiased sample from a randomized trial (see 16-iii).

subitem not at all important

1 (selected)

2

3

4

5

essential

Clear selection

Does your paper address subitem 18-i?

Copy and paste relevant sections from the manuscript (include quotes in quotation marks "like this" to indicate direct quotes from your manuscript), or elaborate on this item by providing additional information not in the ms, or briefly explain why the item is not applicable/relevant for your study

This was not applicable for our trial.

19) All important harms or unintended effects in each group

(for specific guidance see CONSORT for harms)

Does your paper address CONSORT subitem 19? *

Copy and paste relevant sections from the manuscript (include quotes in quotation marks "like this" to indicate direct quotes from your manuscript), or elaborate on this item by providing additional information not in the ms, or briefly explain why the item is not applicable/relevant for your study

Because this was a trial of an animated video series, there was no risk of harms or unintended effects.

19-i) Include privacy breaches, technical problems

Include privacy breaches, technical problems. This does not only include physical “harm” to participants, but also incidents such as perceived or real privacy breaches [1], technical problems, and other unexpected/unintended incidents. “Unintended effects” also includes unintended positive effects [2].

subitem not at all important

- 1 (selected)
- 2
- 3
- 4
- 5

essential

Clear selection

Does your paper address subitem 19-i?

Copy and paste relevant sections from the manuscript (include quotes in quotation marks "like this" to indicate direct quotes from your manuscript), or elaborate on this item by providing additional information not in the ms, or briefly explain why the item is not applicable/relevant for your study

Since we did not develop an app or website, there was no potential for digital privacy breaches.

19-ii) Include qualitative feedback from participants or observations from staff/researchers

Include qualitative feedback from participants or observations from staff/researchers, if available, on strengths and shortcomings of the application, especially if they point to unintended/unexpected effects or uses. This includes (if available) reasons for why people did or did not use the application as intended by the developers.

subitem not at all important

1 (selected)

2

3

4

5

essential

Clear selection

Does your paper address subitem 19-ii?

Copy and paste relevant sections from the manuscript (include quotes in quotation marks "like this" to indicate direct quotes from your manuscript), or elaborate on this item by providing additional information not in the ms, or briefly explain why the item is not applicable/relevant for your study

We did not include this kind of feedback since we did not develop and app or website in this trial.

DISCUSSION

22) Interpretation consistent with results, balancing benefits and harms, and considering other relevant evidence

NPT: In addition, take into account the choice of the comparator, lack of or partial blinding, and unequal expertise of care providers or centers in each group

22-i) Restate study questions and summarize the answers suggested by the data, starting with primary outcomes and process outcomes (use)

Restate study questions and summarize the answers suggested by the data, starting with primary outcomes and process outcomes (use).

subitem not at all important

1 ○

2 ○

3 ○

4 ○

5 ◉

essential

Clear selection

Does your paper address subitem 22-i? *

Copy and paste relevant sections from the manuscript (include quotes in quotation marks "like this" to indicate direct quotes from your manuscript), or elaborate on this item by providing additional information not in the ms, or briefly explain why the item is not applicable/relevant for your study

We clearly restate study questions and summarize the answers suggested by the data in our discussion section.

22-ii) Highlight unanswered new questions, suggest future research

Highlight unanswered new questions, suggest future research.

subitem not at all important

1 ○

2 ○

3 ○

4 ○

5 ◉

essential

Clear selection

Does your paper address subitem 22-ii?

Copy and paste relevant sections from the manuscript (include quotes in quotation marks "like this" to indicate direct quotes from your manuscript), or elaborate on this item by providing additional information not in the ms, or briefly explain why the item is not applicable/relevant for your study

We clearly state in both the Abstract and the Discussion section, the need for more research of this kind in the Global South (in under-resourced settings).

20) Trial limitations, addressing sources of potential bias, imprecision, and, if relevant, multiplicity of analyses

20-i) Typical limitations in ehealth trials

Typical limitations in ehealth trials: Participants in ehealth trials are rarely blinded. Ehealth trials often look at a multiplicity of outcomes, increasing risk for a Type I error. Discuss biases due to non-use of the intervention/usability issues, biases through informed consent procedures, unexpected events.

subitem not at all important

1 (selected)

2

3

4

5

essential

Clear selection

Does your paper address subitem 20-i? *

Copy and paste relevant sections from the manuscript (include quotes in quotation marks "like this" to indicate direct quotes from your manuscript), or elaborate on this item by providing additional information not in the ms, or briefly explain why the item is not applicable/relevant for your study

This was not an eHealth trial in the usual sense of the word, since we did not develop and app or a website.

21) Generalisability (external validity, applicability) of the trial findings

NPT: External validity of the trial findings according to the intervention, comparators, patients, and care providers or centers involved in the trial

21-i) Generalizability to other populations

Generalizability to other populations: In particular, discuss generalizability to a general Internet population, outside of a RCT setting, and general patient population, including applicability of the study results for other organizations

subitem not at all important

1 ○

2 ○

3 ○

4 ○

5 ◉

essential

Clear selection

Does your paper address subitem 21-i?

Copy and paste relevant sections from the manuscript (include quotes in quotation marks "like this" to indicate direct quotes from your manuscript), or elaborate on this item by providing additional information not in the ms, or briefly explain why the item is not applicable/relevant for your study

In our Discussion section, we discuss potential uses for this video series and how it could be adapted for different audiences.

21-ii) Discuss if there were elements in the RCT that would be different in a routine application setting

Discuss if there were elements in the RCT that would be different in a routine application setting (e.g., prompts/reminders, more human involvement, training sessions or other co-interventions) and what impact the omission of these elements could have on use, adoption, or outcomes if the intervention is applied outside of a RCT setting.

subitem not at all important

1 (selected)

2

3

4

5

essential

Clear selection

Does your paper address subitem 21-ii?

Copy and paste relevant sections from the manuscript (include quotes in quotation marks "like this" to indicate direct quotes from your manuscript), or elaborate on this item by providing additional information not in the ms, or briefly explain why the item is not applicable/relevant for your study

Not applicable since we did not develop and app or a website.

OTHER INFORMATION

23) Registration number and name of trial registry

Does your paper address CONSORT subitem 23? *

Copy and paste relevant sections from the manuscript (include quotes in quotation marks "like this" to indicate direct quotes from your manuscript), or elaborate on this item by providing additional information not in the ms, or briefly explain why the item is not applicable/relevant for your study

YEs, we clearly state the trial registry number and date after the Abstract.

24) Where the full trial protocol can be accessed, if available

Does your paper address CONSORT subitem 24? *

Cite a Multimedia Appendix, other reference, or copy and paste relevant sections from the manuscript (include quotes in quotation marks "like this" to indicate direct quotes from your manuscript), or elaborate on this item by providing additional information not in the ms, or briefly explain why the item is not applicable/relevant for your study

We cite the trial protocol, which was published, in the study.

25) Sources of funding and other support (such as supply of drugs), role of funders

Does your paper address CONSORT subitem 25? *

Copy and paste relevant sections from the manuscript (include quotes in quotation marks "like this" to indicate direct quotes from your manuscript), or elaborate on this item by providing additional information not in the ms, or briefly explain why the item is not applicable/relevant for your study

We clearly state the sources of funding in the Acknowledgements section.

X27) Conflicts of Interest (not a CONSORT item)

X27-i) State the relation of the study team towards the system being evaluated

In addition to the usual declaration of interests (financial or otherwise), also state the relation of the study team towards the system being evaluated, i.e., state if the authors/evaluators are distinct from or identical with the developers/sponsors of the intervention.

subitem not at all important

1 ○

2 ○

3 ○

4 ○

5 ◉

essential

Clear selection

Does your paper address subitem X27-i?

Copy and paste relevant sections from the manuscript (include quotes in quotation marks "like this" to indicate direct quotes from your manuscript), or elaborate on this item by providing additional information not in the ms, or briefly explain why the item is not applicable/relevant for your study

We clearly state all study team roles in the methods section.

About the CONSORT EHEALTH checklist

As a result of using this checklist, did you make changes in your manuscript? *

○ yes, major changes

○ yes, minor changes

◉ no

What were the most important changes you made as a result of using this checklist?

We had already completed a CONSORT checklist for this study, so this felt like a repeat.

How much time did you spend on going through the checklist INCLUDING making changes in your manuscript *

This took more than 2 hours.

As a result of using this checklist, do you think your manuscript has improved? *

- ○ yes
- ◉ no
- ○ Other:

Would you like to become involved in the CONSORT EHEALTH group?

This would involve for example becoming involved in participating in a workshop and writing an "Explanation and Elaboration" document

- ○ yes
- ◉ no
- ○ Other:

Clear selection

Any other comments or questions on CONSORT EHEALTH

Your answer

STOP - Save this form as PDF before you click submit

To generate a record that you filled in this form, we recommend to generate a PDF of this page (on a Mac, simply select "print" and then select "print as PDF") before you submit it.

When you submit your (revised) paper to JMIR, please upload the PDF as supplementary file.

Don't worry if some text in the textboxes is cut off, as we still have the complete information in our database. Thank you!

Final step: Click submit !

Click submit so we have your answers in our database!

Submit Clear form

Never submit passwords through Google Forms.

This form was created outside of your domain. Report Abuse - Terms of Service - Privacy Policy

Google Forms
